# Phages are unrecognized players in the ecology of the oral pathogen *Porphyromonas gingivalis*

**DOI:** 10.1186/s40168-023-01607-w

**Published:** 2023-07-25

**Authors:** Cole B. Matrishin, Elaine M. Haase, Floyd E. Dewhirst, Jessica L. Mark Welch, Fabiola Miranda-Sanchez, Tsute Chen, Donald C. MacFarland, Kathryn M. Kauffman

**Affiliations:** 1 Department of Oral Biology, School of Dental Medicine, The University at Buffalo, Buffalo, NY USA; 2 Department of Microbiology, The Forsyth Institute, Cambridge, MA USA; 3 Department of Oral Medicine, Infection and Immunity, Harvard School of Dental Medicine, Boston, MA USA; 4 Department of Pathology and Anatomical Sciences, Jacobs School of Medicine, The University at Buffalo, Buffalo, NY USA

**Keywords:** *Porphyromonas gingivalis*, Phages, Bacteriophages, Oral, Periodontal disease

## Abstract

**Background:**

*Porphyromonas gingivalis* (hereafter “*Pg*”) is an oral pathogen that has been hypothesized to act as a keystone driver of inflammation and periodontal disease. Although *Pg* is most readily recovered from individuals with actively progressing periodontal disease, healthy individuals and those with stable non-progressing disease are also colonized by *Pg*. Insights into the factors shaping the striking strain-level variation in *Pg*, and its variable associations with disease, are needed to achieve a more mechanistic understanding of periodontal disease and its progression. One of the key forces often shaping strain-level diversity in microbial communities is infection of bacteria by their viral (phage) predators and symbionts. Surprisingly, although *Pg* has been the subject of study for over 40 years, essentially nothing is known of its phages, and the prevailing paradigm is that phages are not important in the ecology of *Pg*.

**Results:**

Here we systematically addressed the question of whether *Pg* are infected by phages—and we found that they are. We found that prophages are common in *Pg*, they are genomically diverse, and they encode genes that have the potential to alter *Pg* physiology and interactions. We found that phages represent unrecognized targets of the prevalent CRISPR-Cas defense systems in *Pg*, and that *Pg* strains encode numerous additional mechanistically diverse candidate anti-phage defense systems. We also found that phages and candidate anti-phage defense system elements together are major contributors to strain-level diversity and the species pangenome of this oral pathogen. Finally, we demonstrate that prophages harbored by a model *Pg* strain are active in culture, producing extracellular viral particles in broth cultures.

**Conclusion:**

This work definitively establishes that phages are a major unrecognized force shaping the ecology and intra-species strain-level diversity of the well-studied oral pathogen *Pg*. The foundational phage sequence datasets and model systems that we establish here add to the rich context of all that is already known about *Pg*, and point to numerous avenues of future inquiry that promise to shed new light on fundamental features of phage impacts on human health and disease broadly.

Video Abstract

**Supplementary Information:**

The online version contains supplementary material available at 10.1186/s40168-023-01607-w.

## Introduction

One of the most actively studied and thoroughly described microbial ecosystems is that of the human mouth [1]. A major insight that has emerged from studies of the oral microbiome is that microbially mediated oral inflammation is associated with increased risk for inflammatory disease throughout the body. Understanding the mechanisms underlying the most common oral inflammatory disease, periodontal disease, therefore has relevance not only to oral health but also to systemic health.

*Porphyromonas gingivalis* (hereafter “*Pg*”) is hypothesized to be a keystone pathogen in oral inflammation [2], driving conditions that favor periodontitis, a severe form of periodontal disease. This gram-negative asaccharolytic anaerobe is adapted for growth in the gingival crevice; it colonizes microbial biofilms through attachments to specific bacteria, and its growth is facilitated by growth factors produced by other community members [3]. Once in the gingival crevice, *Pg* has the capacity to produce copious amounts of proteolytic enzymes that facilitate immune evasion and supply its preferred peptide nutrient source. Importantly, *Pg*’s proteolytic activity also contributes to loss of junctional epithelium attachment to the tooth surface, promoting formation of “pockets” of exposed epithelium around the teeth [4], creating niches that favor the growth of other oral opportunistic pathogens, and accelerating disease progression. Notably however, although *Pg* is most readily recovered from individuals with actively progressing periodontal pockets, healthy individuals and those with stable non-progressing pockets are also colonized by *Pg* [5]. Identifying the factors that shape strain-level variation in the physiology, interactions, and virulence potential of *Pg* is thus an important element of achieving a comprehensive view of the role of this species in oral inflammation and systemic disease.

One of the key forces often shaping strain-level diversity in microbial communities is infection of bacteria by viral predators and symbionts, called bacteriophages (hereafter “phages”). Phages are highly specific in the bacterial strains they can infect and replicate in (their host range), in part due to their need to bind to specific host bacterial surface moieties (e.g., lipopolysaccharide, capsule, or outer membrane proteins [6]) in a lock-and-key fashion. As a result, infection and killing by phages can exert a strong diversifying effect on microbial populations through negative frequency dependent selection (favoring rarer strains and genes) [7, 8]. In the oral microbiome, phages are believed to be numerous and diverse, with counts estimated at up to 10^8^ ml^−1^ in saliva and 10^10^ g^−1^ dental plaque [9]. Metagenomic studies of oral viromes have revealed the presence of phage-encoded virulence factors (e.g., prophage genes encoding proteins promoting bacterial binding to platelets [10]), enrichment for genes predicted to shape bacterial interactions with human host cells (e.g., phage genes for secreted ankyrin-repeat domain containing proteins that reduce phagocytosis [11]), and shifts in phage communities in periodontal disease [12]. Elegant early laboratory studies of the processes underlying oral biofilm community assembly also have revealed that the receptors used by oral phages include the very cell surface molecules key to co-evolved coaggregation between different bacterial species (e.g., *Actinomyces* and *Streptococcus* spp.) [13–16]. This latter work suggests that selection pressure exerted by phages is a major factor shaping dynamics of oral biofilm development in vivo. However, despite the potential for studies of phage-bacteria interactions to shed light on the ecology of specific bacteria and the structure and function of microbial communities, cultivated bacteria-phage model systems are lacking for all but a few species in the oral microbiome [17, 18].

The extent to which the key oral pathogen *Pg* interacts with phages remains a major open question. Surprisingly, although *Pg* has been the subject of study for over 40 years [19], essentially nothing is known of its phages, and the prevailing paradigm is that phages are not important in the ecology of *Pg*. An early study [20] using a culture-based approach to uncover prophage interactions in *Pg* found no evidence of plaque formation (lysis of bacteria in lawns), and though future studies with modified approaches were recommended, there is no evidence they were carried out. More recent studies using comparative genomic analyses of *Pg* genomes have identified candidate phage genes [21], and two *Pg* genomes have been noted as having candidate prophage regions, though these are not further described (ATCC 49417, with a region noted as Bacteriophage phi Pg1 in GenBank record FUFH01000018.1; and WW2952, with a candidate prophage noted as PgSL1 [22]). Investigation of the targets of the highly prevalent CRISPR-Cas defense systems in *Pg* have also identified candidate phage genes in *Pg* genomes as potential targets [23]. Yet, because it is thought that there are no phages infecting *Pg* [23], studies of these systems have highlighted other roles [24], finding them to be independently associated with virulence [25] and highly expressed in periodontal disease [26]. The presence of CRISPR-Cas systems in *Pg* genomes has been suggested to explain the lack of phages infecting this species [23]. However, the existence and prevalence of defense systems also implies that selection to maintain these may be due to predation pressure by as-yet-unrecognized phages infecting *Pg*.

Here, we sought to systematically address the question of whether *Pg* are, or are not, infected by phages—and we found that they are. Using a bioinformatic approach, we showed that integrated phages (“prophages”) are common in *Pg*, represent three new proposed family-level groups, and encode genes that have the potential to alter their host physiology and interactions. We found that these phages represent previously unrecognized targets of the prevalent CRISPR-Cas defense systems in *Pg* and that *Pg* strains encode numerous additional mechanistically diverse candidate anti-phage defense systems. We also showed that phages and anti-phage defense system elements together are major contributors to strain-level diversity and the species pangenome of this oral pathogen. Finally, we found that nuclease-protected phage genomes and virus-like particles are present in culture supernatants of a *Pg* strain encoding a prophage. In sum, this work reveals that interactions with phages are a major unrecognized force shaping the ecology and intra-species strain-level diversity of the well-studied oral pathogen *Pg*.

## Results

### *Pg* isolates harbor phylogenetically diverse prophages

To address the question of whether *Pg* interacts with phages, we focused our investigation on temperate phages, which have the capacity to integrate into their host bacterial genomes and form stable associations as prophages. Phages with temperate life history strategies are known to be common in the oral microbiome [27] and offer the possibility of discovery based on study of bacterial genome sequences alone.

Definitive identification of prophages in bacterial genomes remains a challenge for the field. To systematically search for prophages in *Pg* genomes, we therefore used an approach that combined multiple complementary lines of evidence (Supplementary Fig. 1, and see “Materials and methods” for details). In brief, we first analyzed the species pangenome to identify variable genomic regions not present in all *Pg* and thus likely to include mobile elements such as phages. Next, we applied a panel of well-developed prophage prediction and annotation tools to identify the subset of variable genome regions likely to be prophages. We then harvested all CRISPR spacers from identified arrays in *Pg*, as well as other species of bacteria, and mapped these back to all *Pg* genomes to facilitate detection of regions that are likely to be actively mobilizing. Finally, we identified precise boundaries of prophage regions by manual curation using all-by-all BLAST-based genomic comparisons among all *Pg* genomes, all available phage and bacterial annotations, and likely insertion site sequences and bounding repeats. Together, these methods yielded comprehensive and high-quality prophage predictions.

**Fig. 1:**
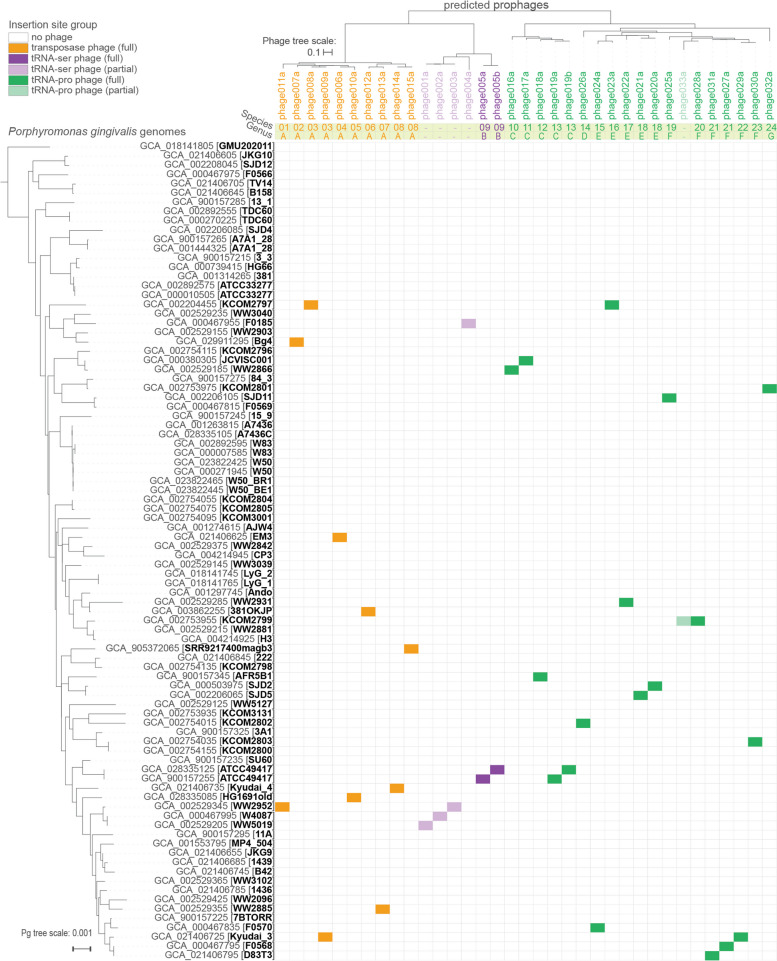
Prophages are common in sequenced *Porphyromonas gingivalis* isolates. Phylogenetic relationships among *Pg* shown on the left (79 strains; 88 leaves, including 3 substrains and 6 strains with independent assemblies), based on concatenated ribosomal protein genes. Relationships among *Pg* phages shown in midpoint-rooted tree at the top (30 full, 5 partial; “b” suffix indicates version of an “a” phage found in a different assembly of the *Pg* strain), based on whole genome nucleotide BLAST distance and scaled by VICTOR [28] d0 formula (recommended for nucleic acid datasets). Candidate genus- and species-level clusters are shown for full-length phages in the yellow bars. Three higher-order clades of phages defined by distinct insertion sites in host genomes (by full-length phages only) are highlighted (see color legend). Colored cells in the matrix indicate the assemblies in which each phage was found

Using our integrative approach, we found that prophages are common in strains of *Pg*, present in 32% (25/79) of strains examined (Fig. 1). We searched for prophages in all publicly available *Pg* genomes, as well as in four additional genomes we sequenced for this work (a total of 79 strains, hereafter “*Pg*_set_79”, and 88 genomes including cases of substrains and re-sequenced strains, hereafter “*Pg*_set_88”; see Supplementary Data 1). Four of the 25 *Pg* strains with prophages encoded two prophages each, and an additional four *Pg* harbored partial prophage regions. We also identified an additional *Pg* with a prophage likely incomplete only due to an assembly artifact (phage033a).

The distribution of prophages with respect to the *Pg* phylogeny did not reveal obvious patterns of association with specific clades in this dataset (Fig. 1), nor suggest host ranges dependent on use of potential receptors linked with virulence (e.g., *mfa*, *rag*, and *fim* gene alleles or K-/O-antigen types, to the extent they are known; see Supplementary Data 2). However, an exploratory analysis of metagenomes from a previous study [29], prepared from subgingival plaque of 6 healthy individuals and 7 with periodontitis, revealed an apparent phage bloom in one of the periodontitis cases, with > 2% of reads mapping to phage012 (Supplementary Fig. 2).

To understand the relationships of the *Pg* phages to previously characterized phages, we used a stepwise approach to ultimately identify them as representing three new candidate family-level units and associated new candidate genera and species. In brief, we clustered the phages with a large collection of reference phage sequences (4912) using vConTACT2 [30] and the ViPTree [31] Virus-Host DB [32] reference database (Supplementary Fig. 3), identified nearest neighbors using ViPTree [31] (Supplementary Fig. 4), resolved family-level units on the basis of shared protein clusters using VirClust [33] (Supplementary Fig. 5) and whole proteome intergenomic distances using VICTOR [28] (Supplementary Fig. 6), and resolved genus- and species-level units on the basis of whole genome nucleotide similarity using VIRIDIC [34]; see “Materials and methods” for details and Supplementary Data 3 for associated data. Phages in these three major groups differed in their genome organization and overall protein content (Supplementary Fig. 7), as well as in their use of different insertion sites in their *Pg* host genomes. One group [present in 10 *Pg* strains], which we propose to name *Alisviridae*, is characterized by non-site-specific transposition-based insertion into the host genome, a feature shared by the unclassified cosmopolitan and broad host range *Bacteroides dorei* phage Hankyphage p00 [35] that is the only reference phage in this new candidate family. This trait is also shared by *Flavobacteriales* phages in the nearest named viral family, the *Winoviridae* [36]. The second group [in 5 *Pg* strains], which we propose to name *Ludisviridae*, is characterized by insertion into the host’s tRNA-serine gene, a feature shared by temperate relatives of the unclassified reference *Riemerella anatipestifer* phage RAP44 [37, 38] that is a member of this candidate family. The third group [in 17 *Pg* strains], which we propose to name *Nixviridae*, is characterized by insertion into the host’s tRNA-proline gene, and includes no previously characterized reference phages.

To also investigate their relationships to phages in other bacterial genomes and metagenomes, we clustered the *Pg* phages with all Uncultivated Viral Genomes (UViGs) in IMG/VRv4 [39] that were predicted to have hosts in the *Porphyromonadaceae* (1138), using vConTACT2 [30] (Supplementary Fig. 8). UViGs in IMG/VRv4 [39] are identified by geNomad [40], a newly developed phage prediction tool, released during the course of our preparation of this manuscript. Investigating UViGs clustering with each of the three *Pg* phage candidate families (Supplementary Figs. 9, 10 and 11), we find these include geNomad [40] UViG versions of some prophages identified and curated in our study (14/28 complete phages identified in our study have geNomad-based UViG counterparts); UViGs representing phages predicted to infect *Porphyromonas gulae* (a sister clade to *Pg* found in dogs), including an example of a *P. gulae* phage in the same genus-level group as *Pg* phages; and UViGs derived from oral and intestinal metagenomes and representing distinct genus-level groups within the candidate *Pg* phage families.

### *Pg* phage genomes harbor genes with potential to shape host ecology

**Fig. 2:**
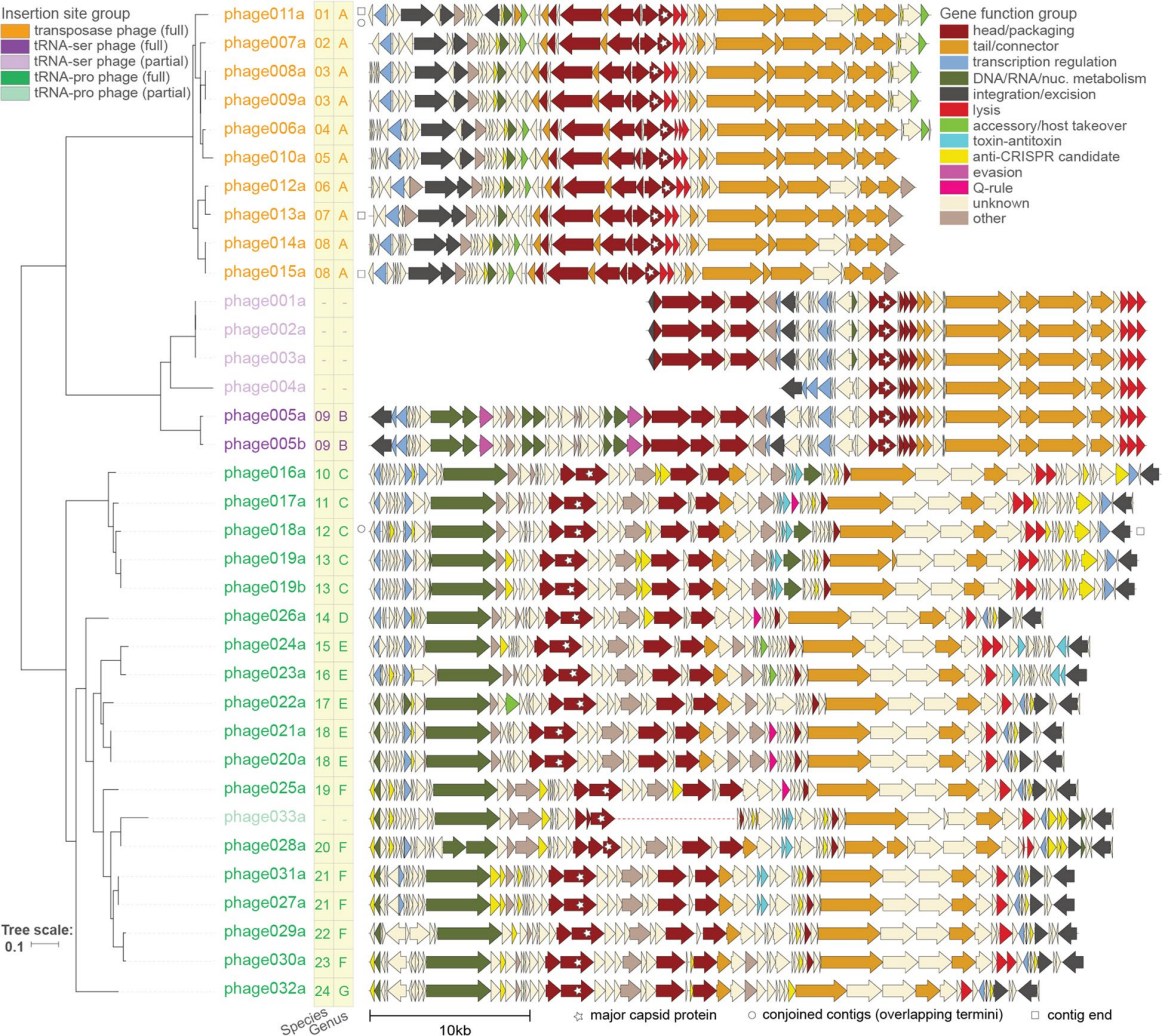
Genome diagrams of *Porphyromonas gingivalis* phages highlight functional annotations and gene order conservation in three large clades defined by distinct use of host genome insertion sites. Representations of *Pg* phage genomes (30 full, 5 partial; names of full-length phages are in saturated colors and partial phages are in lighter shades; “b” suffix indicates version of an “a” phage found in a different assembly of the *Pg* strain), generated using Clinker [42] and showing predicted protein-coding genes as block arrows colored based on predicted protein functional categories (see Supplementary Fig. 7 for version with protein clustering). Relationships among *Pg* phages shown in midpoint-rooted tree at left, based on whole genome nucleotide BLAST distance and scaled by VICTOR [28] d0 formula (recommended for nucleic acid datasets). Candidate genus- and species-level clusters are shown for full-length phages in the yellow bars. Three higher-order clades of phages defined by distinct insertion sites in host genomes (by full-length phages only) are highlighted by coloring of phage names (orange: transposition-based insertion; purple: tRNA-ser; green: tRNA-pro). White stars mark phage genome ends defined by contig ends, circles mark phage genomes identified in this work by joining contigs with overlapping termini, the dotted line in the middle of phage033a highlights that this phage was identified at the two termini of a bacterial contig assembly and is missing genes potentially due to an incomplete assembly

To understand the potential impacts of phages on *Pg* hosts that they infect, we used numerous annotation databases and iterative HMM-based searches to predict the functions of their genes (see “Materials and methods,” Supplementary Data 4). In general, the phage genes most readily annotated are those encoding structural and packaging components of the virion (e.g., capsid, tail, portal, terminase large subunit), and this held true for the *Pg* phages (Fig. 2). Based on sequence similarity and conservation of structural gene order [41], all phages identified here were predicted to be siphoviruses with long non-contractile tails. However, predicted structural and assembly genes together accounted for only 363 (19%) of the 1892 genes in these 33 phages, and the majority of phage genes (60%) could not be readily annotated (Supplementary Data 4, excluding “b” versions of phages, which represent the same phage region as the “a” version in a different assembly of the same host strain).

Despite the challenges of annotating phage genes, several intriguing classes of genes with the potential to impact *Pg* physiology and virulence emerged in this first investigation. Among these were genes encoding (1) putative lipopolysaccharide (LPS)-modifying enzymes, (2) proteins with signal peptides targeting them to transport by the general secretion system, and (3) toxin-antitoxin systems; we highlight these examples below.

First, the majority of phages in the proposed family *Alisviridae*, characterized by transposition-based insertion, encode putative phosphoheptose isomerases, genes that participate in LPS synthesis (light green genes in orange phage group in Fig. 2, Supplementary Data 4). The presence of LPS-modifying genes in phages has previously been shown to result in modifications of host bacterial LPS that alter bacterial virulence potential and sensitivity to infection by related phages [43, 44]. That this gene is common in the transposable phage clade suggests that LPS may be a receptor for this group, as it is for the transposable phage Mu [45]. This finding points to transposable phages having the potential to alter *Pg* ecology and virulence not only through inactivation of genes upon non-specific integration into bacterial genomes, but also through modification of host LPS, a key contributor to *Pg* virulence.

Second, multiple phages in the proposed family *Nixviridae*, those inserting into tRNA-pro genes, encode genes with signal peptide sequences. A subset of these genes encode proteins associated with core phage functions, including major capsid proteins whose signal peptides are likely cleaved by the phage-encoded prohead proteases, and spanins, which are necessary for lysis. Yet strikingly, five genes of unknown function with signal peptides are predicted to obey the “*Bacteroidetes* Q Rule” [46], whereby cleavage of the signal peptide is predicted to expose an N-terminal glutamine residue (neon pink genes in green phage group in Fig. 2, Supplementary Data 4). The Q-rule is a unique and distinctive feature of Signal Peptidase I substrates in the *Bacteroidetes* [46]. That *Pg* phages encode proteins that follow this rule suggests that they are adapted to using their host’s general secretion systems and have the potential to modify *Pg* outer membranes and thereby their interactions.

Third, phages in the proposed family *Nixviridae* also commonly encode toxin-antitoxin (TA) system genes (neon blue genes in green phage group in Fig. 2, Supplementary Data 4). TA systems are mechanistically diverse but share the property of encoding a toxin that reduces bacterial metabolic activity and an antitoxin that neutralizes the toxin. These systems are upregulated in bacteria as defenses in response to phage infection [47, 48], and as a survival strategy during other cellular stress events; for example, TA systems can induce a persister state upon exposure to antibiotics or nutrient starvation [49]. Although TA systems encoded on phages may play a role in promoting maintenance of these selfish elements in their host populations, they have also recently been shown to act in inter-phage competition [50], preventing successful infections of the host by other phages. The diverse roles of TAs in bacterial physiology raise the question of whether *Pg* prophages encoding TAs can provide an ecological advantage to their hosts in the stressful subgingival crevice [51]. The most readily recognizable TA systems in the *Pg* phages are Type II HicAB dyads that function by degrading mRNA, reversibly reducing global translation [51, 52]. Additional singleton toxins and antitoxins are also present in the prophages. Solo antitoxin genes encoded in phage genomes have been shown to act as counter-defenses to bacterial TA-mediated attempts to abort infections [53]. Solo toxins, however, are not expected, and as we found these in genomic islands, known to be used by phages to harbor anti-phage genes [50], we expect that partner genes for these singletons will ultimately be identified among nearby genes. Future studies examining expression of integrated prophage TA genes in *Pg* strains across physiologically relevant growth conditions (including exposure to predation by exogenous phages) are needed to reveal whether they play a role in promoting *Pg* survival.

### Prophages are targets of *Pg* CRISPR systems and encode putative anti-CRISPR genes

**Fig. 3:**
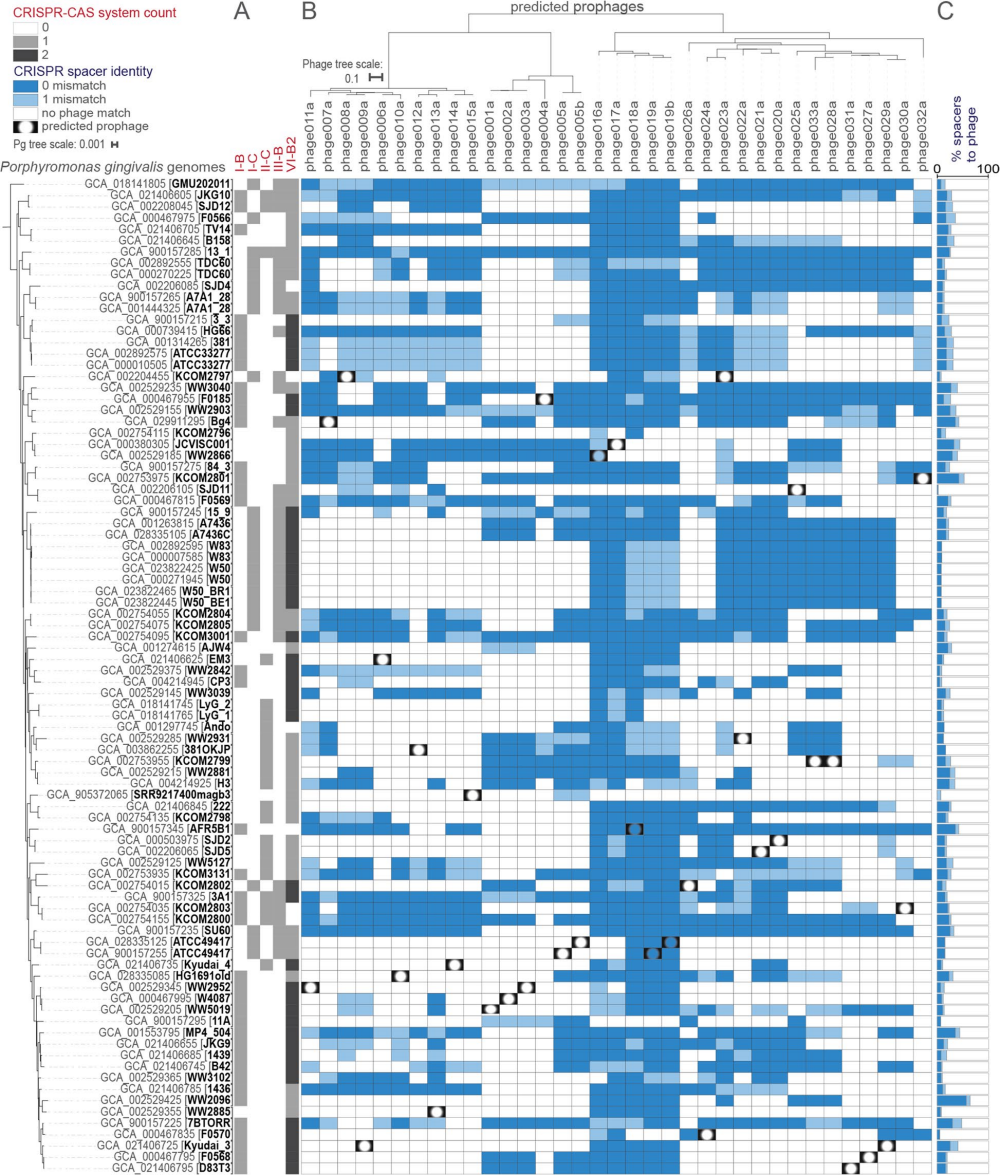
*Porphyromonas gingivalis* CRISPR arrays encode spacers that target phages in other strains. Predicted CRISPR-Cas systems in each strain of *Pg* are shown; quantities of each system are related to cell color saturation (A). CRISPR spacer hits from arrays found in *Pg* are mapped onto *Pg* phages shown in midpoint-rooted tree at the top (30 full, 5 partial; “b” suffix indicates version of an “a” phage found in a different assembly of the *Pg* strain (based on whole genome nucleotide BLAST distance and scaled by VICTOR [28] d0 formula, recommended for nucleic acid datasets) dark blue cells indicate 0-mismatch spacer-phage nucleotide identity, light blue indicates 1-mismatch, and vignetting indicates presence of the entire prophage in the bacteria (as shown in Fig. 1) (B). Percent of total spacers found in each *Pg* that have 0- or 1-mismatch to a predicted phage are shown; same coloring as panel B (C). CRISPR-Cas systems were identified by CCTyper [55] and mapped to phage genomes with Bowtie [56]. Phylogenetic relationships among *Pg* are shown on the left (79 strains; 88 leaves, including 3 substrains and 6 strains with independent assemblies), based on concatenated ribosomal protein genes

Finding that prophages are common in *Pg* raised the question of whether they represent targets of spacers in *Pg* CRISPR arrays. *Pg* strains commonly encode CRISPR arrays, yet the targets of the spacers have remained elusive [23, 54]. To address the question of potential phage targeting by *Pg* CRISPR systems, we harvested the spacers from *Pg* genomes in our dataset using CRISPRCasTyper [55] (CCTyper) and compared these with the sequences of the prophages (Fig. 3, see “Materials and methods”).

Overall, we found that prophages account for a substantial fraction of targets of CRISPR array spacers in *Pg*. Every *Pg* strain we investigated encodes at least one CRISPR-Cas system (Fig. 3A), and their arrays collectively encode 4993 spacers (*Pg*_set79, see Supplementary Data 5). Considering spacers in all *Pg* genomes, a total of 833 (17%) showed 100% nucleotide identity to at least one of the phages characterized in this study (Fig. 3B), and 1150 (23%) mapped to phages if allowing 1 nucleotide mismatch in the alignment (see Supplementary Data 6). Considering spacers in individual strains of *Pg*, we found that the proportion targeting phages can be far larger; up to 57% of spacers in a given strain were sequence-identical to phages in this collection (up to 64% if allowing 1 mismatch in mapping, Fig. 3C). As we expect that the diversity of *Pg* phages exceeds that which we have captured in this limited number of genomes, we also considered the possibility that spacers whose targets were not yet identified might target phages more distantly related to those in our dataset (e.g., showing protein conservation but nucleotide divergence). To address this possibility, we used SpacePHARER [57] to translate spacer nucleic acid sequences in all 6 reading frames and map these peptides against *Pg* phage proteins. This translation-based mapping increased the proportion of spacers that could be matched to phages in our dataset to 27% (1342/4993 matches, Supplementary Data 7).

As expected, the majority of *Pg* strains do not carry spacers that map to their own prophages, yet we noticed that a small number do (3 of 28 strains with prophages in *Pg*_set79; see filled-in “peepholes” in Fig. 3B). In two cases, there are only few matching spacers, however, strain AFR5B1 appears to extensively target its own phage018a (14 0-mismatch spacer hits from a Class 1 Type I-B array, and 17 spacer hits if allowing up to 1 mismatch, see Supplementary Data 6). The large number of matches to phage018a in AFR5B1 raised the question of whether this phage encodes anti-CRISPR protein genes (phage counter-defense genes protecting against CRISPR-Cas systems), that would have allowed it to survive targeting upon infection to successfully achieve integration [58].

To investigate whether genes encoding anti-CRISPRs (acrs) are present in *Pg* phages, we used tools and databases designed for their discovery, PaCRISPR [59] and the DeepAcr database [60]. This approach yielded numerous candidates; to select those of highest confidence, we considered only those identified by both PaCRISPR and DeepAcr, and in this way identified 99 candidate acr genes (in 26 distinct protein sequence clusters; Supplementary Data 4). Candidate acr genes occurred in variable regions in phage genomes, enriched in small, often hypothetical, genes (Fig. 2, yellow genes). In the genome of phage018a, mentioned above as being heavily targeted by spacers in its own parent *Pg* genome, we found six candidate acrs. Though these predicted acrs require future study for validation, our findings suggest that such genes may indeed have played a role in the successful integration of phage018A into AFR5B1 by inhibiting CRISPR-Cas targeting [58]. *Pg* prophages thus offer plentiful candidate acrs for future in vitro functional validation and characterization of phage genes involved in the bacteria-phage arms race in the human oral microbiome.

We found that the most prevalent CRISPR-Cas systems in *Pg* were the Class 2 Type VI systems (73/79 strains) and, collectively, spacers from these arrays targeted all 24 candidate species of *Pg* phages at 100% identity. Although Type VI systems are generally rare in bacteria [61], and few well-characterized phages have counter-defense mechanisms effective against them [62], these systems are widespread in the *Bacteroidetes* and *Fusobacteria*. The majority of spacers in the *Pg* were, however, encoded by Type I-B rather than Type VI-B arrays (Type I-B: 51%, I-C:17%, II-C: 11%, III-B: 4%, VI-B2: 17%, Supplementary Data 5). That Type VI systems offer broad spectrum activity against phages [62], likely in part due to their lack of a requirement for protospacer associated motifs, suggests the possibility that fewer spacers are needed by these systems to achieve coverage of diverse phages. Whereas Type VI effector genes were commonly identified (Cas13b, and the Cas13b-activated membrane pore-forming Csx28 [63], identified as tm_HEPN by CCTyper [55]), genes associated with the adaptation module (e.g., cas1 or cas2) were not. In some cases, it has been shown that Type VI-B systems can acquire spacers in trans from other co-occurring systems (e.g., from Type II-C systems in *Flavobacterium* [64]); however, we found no spacers or repeats shared between Type VI-B and any other array types in *Pg* (Supplementary Fig. 12, Supplementary Data 5). This suggests that novel Type VI CRISPR-Cas adaptation modules likely lie among the conserved hypothetical genes near Type VI effector genes.

We also took advantage of CRISPR spacers to explore whether these *Pg* phages may have hosts in other bacterial species. To do this, we used CRISPROpenDb [65], which maps > 1.3 million unique spacers harvested from CRISPR arrays in 1978 bacterial genera to potential targets. We found that matches between array spacers in *Porphyromonas gulae* and *Pg* prophages were common (242 100% identity matches, see Supplementary Data 8). These observations are consistent with our finding of *P. gulae* phages as among close relatives of the *Pg* phages (Supplementary Figs. 9 and 11) and suggest that phages infecting these closely related bacterial species have the potential to recombine if they were to co-infect.

Overall, our finding that *Pg* CRISPR-Cas arrays are enriched for spacers that target phages confirms that, in addition to their potential roles in bacterial physiology and virulence [25, 66], defense against phage infection is one of their major functions in this species. The unceasing bacteria-phage arms race [67] is reflected here in the numerous candidate acrs we found in phages. Recent work has highlighted the complexity [68] and specificity [69, 70] of interactions between defense and counter-defense systems and unraveling the structure of these interactions to predict phage host ranges at the bacterial strain level remains a major challenge for the field.

### Non-CRISPR-Cas defense systems are also common and diverse in *Pg* genomes

**Fig. 4:**
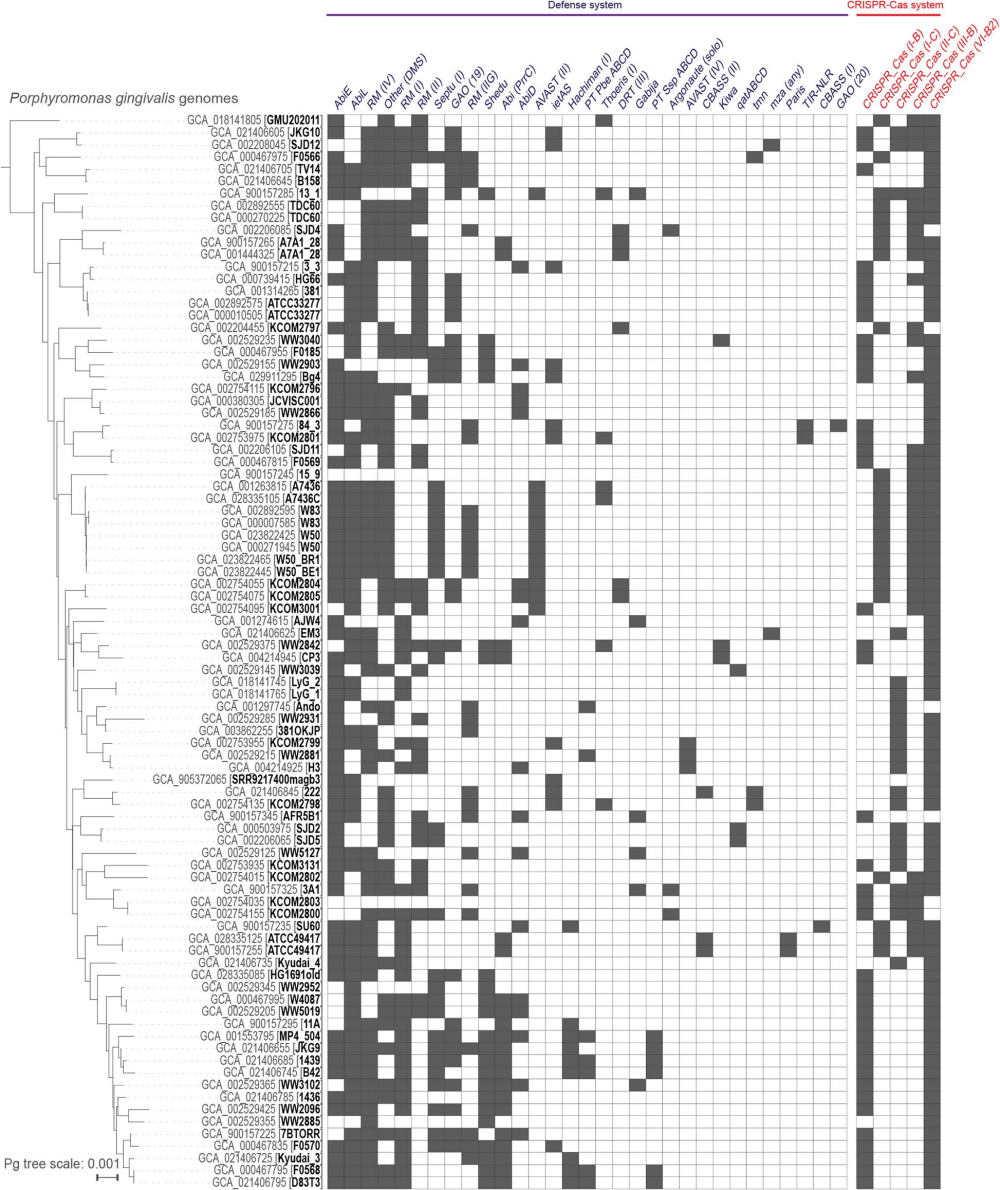
The *Porphyromonas gingivalis* species-level pan-immune system is diverse. Presence of defense systems in each strain of *Pg* are indicated by filled in cells. Systems identified by PADLOC [72] (excluding CRISPR-Cas systems) shown with subtypes indicated within parentheses where applicable, CRISPR-Cas systems identified by CCTyper [55]. Phylogenetic relationships among *Pg* are shown on the left (79 strains; 88 leaves, including 3 substrains and 6 strains with independent assemblies), based on concatenated ribosomal protein genes

Defense systems in bacteria are highly diverse [71], and to expand our investigation of anti-phage defenses beyond CRISPR-Cas systems, we screened all 88 *Pg* genomes for the presence of any of the > 100 systems in PADLOC-DB v1.4 [72]. We found that there are at least 31 non-CRISPR-Cas systems in *Pg* (Fig. 4, Supplementary Data 9), including abortive infections systems, restriction-modification systems, retron-based interference systems (e.g., Septu [73]), and systems that use cyclic nucleotides to activate effectors (e.g., CBASS [74] and Thoeris [73]).

As previously highlighted in the context of the bacteria-phage arms race, “Where there is defense, there is counter-defense” [75]. One example of such a counter-defense system is an immune evasion associated nuclease (Anti-Pycsar, Apyc1) predicted with high confidence in phage005 (purple gene in Fig. 2, gene phage005a_ATCC49417_12 in Supplementary Data 4). Anti-Pycsar nucleases allow phages to escape bacterial immune systems by degrading the cyclic nucleotides that activate defense effectors [76]. That we did not identify a pycsar system in these *Pgs* suggests either that divergent pycsar-like systems are among the hypothetical genes in these strains, that they are present in strains outside this collection, or that anti-pycsar-like nucleases target additional classes of bacterial immune systems. Though in-depth searches for phage counter-defense or immune evasion genes are beyond the scope of this work, the finding of one such system points to the presence of others among the numerous hypothetical genes in *Pg* prophages and this example provides a valuable model system for further characterization of the phage-bacteria arms race in the oral microbiome.

### Prophages and defense-related islands are a major part of the *Pg* pangenome

As differences in carriage of prophages and anti-phage defense systems are a major source of intra-species diversity in other bacteria [69, 71], we next asked to what extent this is also true for *Pg*. Using a pangenome approach that considered conservation of gene ordering rather than numeric prevalence thresholds alone (PPanGGOLiN [77]), we clustered all protein-coding genes to identify genes present in nearly all *Pg* (“core” gene families; > 87% of genomes in this dataset) and genes that are variably present in *Pg* genomes (“flexible” gene families) (see “Materials and methods”). At the level of individual *Pg*, we found that almost a quarter (23%) of every strain’s genome was composed of flexible genes not shared by all members of the species. Across all genomes, together we found 5745 gene families (*Pg*_set_79), with 1476 (26%) core, and the remaining 4269 (74%) flexible (Supplementary Fig. 13 and Supplementary Table 1). This large contribution of the flexible genome to the pangenome of *Pg* is consistent with a previous study that showed that pangenomes of species in the order *Bacteroidales*, including *Pg*, generally have a large contribution of flexible genes (e.g., from 69% in *Odoribacter splanchnicus* to 89% for *Bacteroides vulgatus*, based on summed “shell” and “cloud” gene families) [77].

The curation of phages described in this work allowed us to identify 8% of flexible *Pg* pangenome protein clusters as encoded by prophages (351/4269 gene families, Supplementary Table 1, Supplementary Data 9). To also obtain an estimate of the contribution of defense systems to the *Pg* pangenome, we quantified gene families associated with regions of genome plasticity (PPanGGOLiN [77] “RGPs”, comprised of runs of adjacent flexible genes) encoding either CRISPR-Cas systems (as predicted by CCTyper, Fig. 4) or other defense systems (as predicted by PADLOC [72] and by manual annotation, see “Materials and methods” and Fig. 4). Using this approach, we found that 38% of flexible protein clusters (1636/4269, Supplementary Table 1, Supplementary Data 9) were encoded on likely defense islands. Thus, prophages and putative defense elements together comprise 46% of flexible gene families in the *Pg* pangenome.

Given our systematic curation of prophages in this dataset, we expect our estimate of the relative contribution of prophages to the *Pg* pangenome to remain fairly stable as new *Pg* isolates are sequenced in the future. However, our estimate of the contribution of predicted defense islands to the *Pg* pangenome is likely to be conservative, as it relied on functional annotation of genes and, based on initial Bakta [78] annotations, nearly half of all *Pg* gene families (48%) were of unknown function. Islands of genes related to defense against phages are known to be major contributors to strain-level diversity in environmental bacteria [69, 71, 73], and with further study, numerous additional gene families of currently unknown function in *Pg* will likely be revealed as defense systems. Altogether, these findings demonstrate that prophages, and the defense systems that protect against them, are important contributors to strain-level diversity in *Pg*.

Although we focused on the phages and defense systems for this work, transposons were also notably prominent in the pangenome. In particular, Insertion Sequence (IS) transposases were highly diverse (represented by at least 3448 genes in 61 protein clusters, see Supplementary Data 10) and abundant, with some genomes having as many as 117 transposases. These findings are consistent with previous studies that have established IS elements as highly abundant and diverse in *Pg* [79], contributing to gene regulation [80] and genome recombination and targeted by CRISPR systems [24]. Notably, one of the IS elements that we identified in this work was found to be present in a transposable prophage (phage011a). Sequence comparisons revealed that the IS element present on the phage had > 98% sequence identity over its entire length to elements in *Pg* strains Bg4, KCOM2797, and Kyudai3, but not to any IS’s in its own host, WW2952. This case suggests the possibility that the IS elements so ubiquitous in *Pg* genomes may be hitching rides on phages, using them as vectors of horizontal gene transfer, and benefiting from the phage’s capacity for immune evasion and counter-defense.

### Prophages in *Pg* are active in culture

**Fig. 5:**
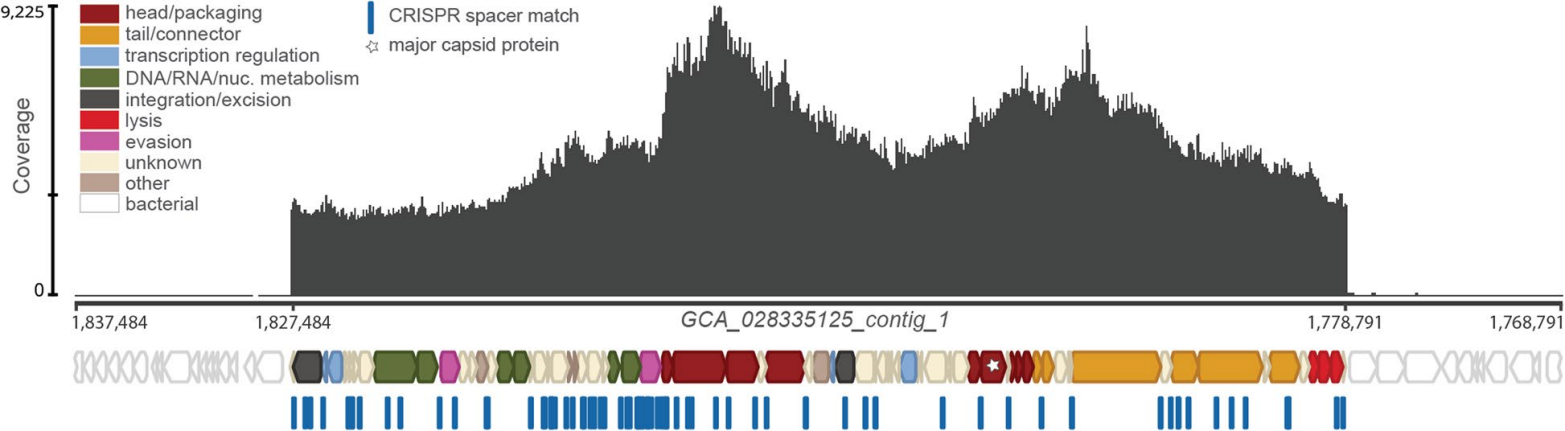
Protected phage DNA is present in *Pg* cultures. Coverage (dark gray plot) of nuclease-protected DNA sequences from a filtered 20-day-old ATCC 49417 culture mapped onto a section of the ATCC 49417 genome that was assembled from the same untreated, 1-day-old culture. An ~ 49 kb spike in coverage, with maximum 9225 × coverage (indicated by the scale on the left, middle hash mark notes mean coverage), corresponds with the region of phage005b in GCA_028335125_contig_1. Colored block arrows represent phage genes predicted by Cenote-Taker2 [81] (major capsid protein marked with star), while white block arrows represent host genes predicted by Bakta [78]. CRISPR spacer matches to phage005b found in other *Pg*, predicted by CCTyper [55] and mapped with Bowtie [56] (100% identity), are represented by blue dash marks

**Fig. 6:**
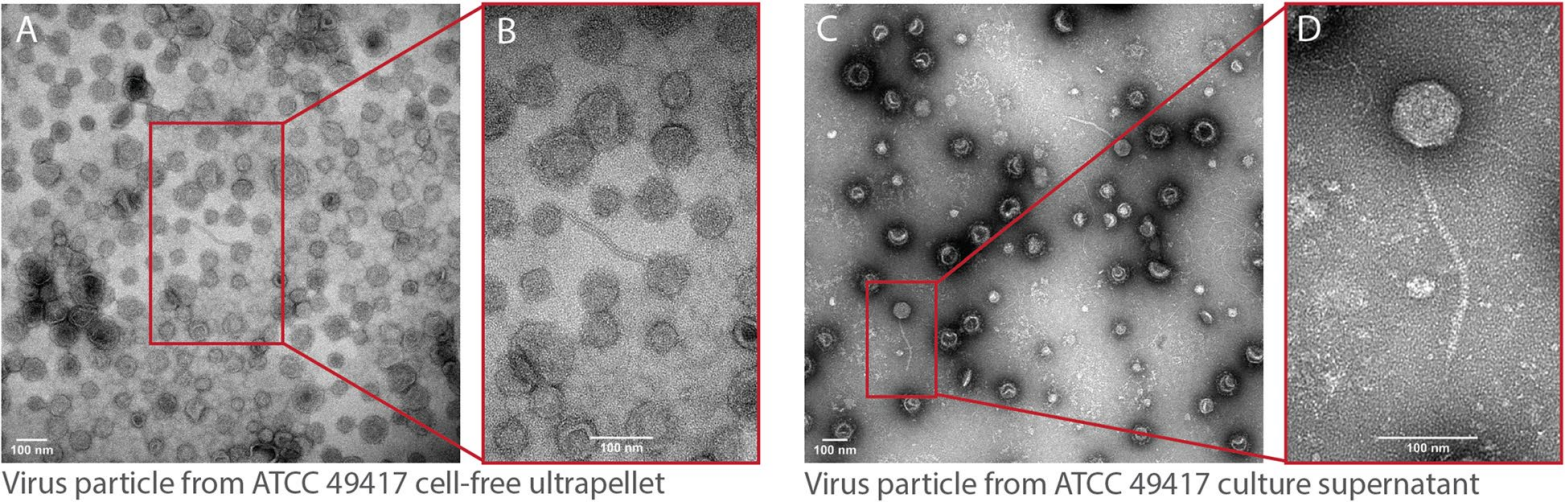
Virus-like particles are produced by *Pg* cultures. Transmission electron micrograph (2.73 pixels/nm) of the ATCC 49417 cell-free, ultracentrifuged supernatant that, when sequenced, produced the reads mapped in Fig. 5; virus-like particles were sparse among likely extracellular vesicles, despite high coverage of the prophage region in DNA from this material (**A**). Magnification (5.52 pixels/nm) of the virus-like particle from panel **A** (**B**). Transmission electron micrograph (2.25 pixels/nm) of supernatants of a 3-day-aged ATCC 49417 culture derived from passages of the same stock that gave rise to the cultures imaged in panels **A** and **B**; virus-like particles were more abundant than in ultracentrifuge pellets shown in panels **A** and **B** and more commonly showed angular, icosahedral capsids (**C**). Magnification (8.81 pixels/nm) of the virus-like particle from panel **C** (**D**)

Finally, to investigate whether there is evidence for activation of *Pg* prophages in culture, we conducted laboratory studies focusing on a model strain (ATCC 49417) predicted to encode two functional prophages. These studies revealed the presence of abundant extracellular, nuclease-protected phage DNA from one of the two phages in culture supernatants (Fig. 5). Aged broth cultures of ATCC 49417 were filtered through 0.2-μm filters to remove bacterial cells and ultracentrifuged at 174,900 × *g* to pellet cell-free particles. Ultracentrifuge pellets were nuclease-treated to remove unprotected DNA prior to extraction, and Illumina sequencing revealed a high coverage enrichment of DNA from the region of the predicted siphovirus phage005, as compared with sequence from the background bacterial chromosome (Fig. 5, Supplementary Fig. 14, Supplementary Data 11). Electron microscopy of material from the resuspended pellet showed highly abundant particles of irregular size (presumably outer membrane vesicles) (Fig. 6A) as well as phage-like particles (Fig. 6B) similar to those observed in previous exploratory imaging studies of the same strain directly from broth cultures (Fig. 6C, D). Together, these observations indicate that cultures of *Pg* encoding prophages can produce cell-free nuclease-protected phage DNA and virus-like particles under common laboratory conditions.

## Discussion

The discovery in this work that *Pg* are commonly infected by phages, and commit significant genomic real estate to predicted defense elements to protect against them, raises many new questions about this well-studied pathogen. Three broad areas of special interest for future studies are highlighted here.

### Dynamics of *Pg* prophage activation

In this work, we showed that a strain of *Pg* harboring prophages naturally releases phage particles and DNA into the supernatant under standard broth culture conditions. Natural release of phages is known in other systems, including the related gut-associated *Bacteroides fragilis* [82]. However, we observed that in a *Pg* host with two prophages, only one phage dominated in the supernatant of broth cultures, and recent work has also shown that where bacterial strains harbor multiple prophages, these can have distinctive induction (activation) profiles [83]. This raises the question of what the natural cues are that different prophages are “listening in on” in vivo in the gingival crevice. Knowledge of which inducers are relevant in the oral microbiome has implications for understanding when there may be increased rates of cell lysis mediated by phage replication, and how different types of phages differentially impact lysis across conditions. Increased cell lysis has the potential to contribute to biofilm development through the release of free DNA, stimulate inflammation through release of bacterial cell debris, and facilitate horizontal gene transfer by multiple mechanisms. Previous studies have shown that a natural inducer in the oropharynx is hydrogen peroxide, which, for example, when produced by *Streptococcus pneumoniae* allows it to displace and outcompete *Staphylococcus aureus* competitors through “remote-control” induction of prophages [84]. A study of the response of *Pg* ATCC 49417 (the strain for which we demonstrated phage release in culture) to hydrogen peroxide [85] showed a culture-medium-dependent inhibitory effect at 3 mM, suggesting that hydrogen peroxide may also play a role as an inducer of *Pg* phages.

Although the majority of phage genomes we identified are complete and encode tails, and we have shown that they can form extracellular tailed-phage particles, the copious production of vesicles by *Pg* also raises the question of whether phages can use vesicles as a mode of transmission. Release of phages in vesicles has been reported [86], and DNA packaged into vesicles by *Pg* ATCC 49417 has been shown to be transferred and expressed in *Pg* ATCC 33277 [87]. Relatedly, we also observed reproducible presence of nuclease-protected non-phage DNA in ATCC 49417 supernatants, suggesting that specific regions of *Pg* genomes are potentially packaged into vesicles and raising the question of whether packaging of non-phage DNA is associated with prophage activation. A recent study [88] that focused on understanding dynamics of one of the most abundant groups of phages in the gut microbiome, the obligately lytic crAssphages infecting the *Bacteroides*, found that they do not form plaques in standard phage assays nor reduce turbidity of broth cultures of their hosts, though they are actively replicating. In general, it appears that phage infection dynamics in human-associated *Bacteroidetes* may commonly diverge from expectations based on studies of canonical model systems (e.g., lambda- and T-phages infecting *E. coli*). Resolving dynamics of prophage activation and spread in *Pg* model systems thus will likely also provide insight into phage-bacteria interactions in the human microbiome generally.

### Impacts of integrated phages on *Pg* physiology

In addition to identifying genes that likely alter *Pg* surface properties, we also showed that *Pg* phages harbor numerous genes of as-yet unknown function. A recent study [89] demonstrated the power of transcriptomics to identify genes expressed by otherwise quiescent prophages, and revealed these as candidate modulators of host physiology across growth conditions (e.g., starvation, exposure to macrophages). Similar studies of new *Pg* phage model systems will be important for achieving a mechanistic understanding of how these phages are impacting *Pg*. In addition, the pressure for *Pg* strains to harbor defense systems against phages may impose fitness costs reflected in trade-offs between sensitivity to phages and growth rate. An elegant study [90] in a marine system showed that the majority of bacteria in a population are resistant to phages yet are also slower growing compared with the rare phage-sensitive strains. To what extent the trade-off between defense and growth holds true for phage-bacteria interactions in the human microbiome is an important open question.

### Role of phages in oral colonization by *Pg* in health and disease

Since the earliest days of their discovery in the 1900s, phages have been recognized for their potential to protect humans from bacterial pathogens, both through natural acquisition from the environment [91] and through exogenous application as “phage therapy” [92]. Recently, phages in the human microbiome have also been shown to bind to human mucins, forming a line of defense against colonization by pathogens [93]. Our finding that *Pg* strains commonly harbor prophages raises the question of whether phages also play a role in intra-species antagonism in this species in the mouth. Such dynamics would have the potential to limit colonization by new strains of *Pg*, either at the level of the person or at the level of individual periodontal pockets, as a result of killing by resident *Pg* phages. Recent work has shown that in the related gut-associated *Bacteroides fragilis*, activated prophages are an important mechanism of intra-species antagonism and cross-killing [82], and in Sandmeier et al.’s [20] search for *Pg* prophages in 1993 [20] they observed antagonism between strains of *Pg*, though this could not be linked to phages. As individuals with *Pg* often harbor multiple strains of the species, with increasing numbers observed in periodontal disease [94], the potential for phage-mediated intra-species antagonism also raises the possibility that bursts of disease progression are the result of bouts of phage-mediated cross-killing. Such a model was proposed in early studies of *Aggregatibacter* phages, where phage activity correlated with local disease progression [95]. Of note, if cross-killing dynamics are an important mechanism of periodontal disease progression, the presence or absence of specific phages alone is not expected to be predictive. Instead, the important property of the system will be the extent to which the specific strains of *Pg* colonizing an individual antagonize one another through phage-mediated mechanisms. That is, the specific structure of phage-bacteria interactions within individual pockets would matter for predicting outcomes. In light of the highly upregulated expression of CRISPR-Cas systems during periodontal disease progression [26], our finding of extensive targeting of phages by *Pg* CRISPR array spacers and our observation of a large phage bloom in a periodontitis patient together suggest that increased phage activity may be an important missed contributor to periodontal disease progression.

Identifying the determinants of host range for *Pg* phages will be an essential next step toward understanding how phages shape interactions between strains of *Pg* and between *Pg* and other microbes and the human host. This will include defining the cell surface receptors used by phages for adsorption and entry, as well as resolving the relationships between specific bacterial defense and phage counter-defense (immune evasion) genes [69, 70]. Defining these receptors is also expected to provide new insights into the selective pressures acting on *Pg* expression of cell surface moieties (e.g., O- and K-antigens, fimbriae, and other outer membrane proteins [6]) commonly used by phages to infect their hosts and that play a role in virulence and capacity for *Pg* to bind to partners in oral biofilms (e.g., *Streptococcus gordonii* [96]), recruit other species (e.g., *Fusobacterium nucleatum* [97]), bind to and invade human host cells, and avoid phagocytosis.

Ultimately, understanding the roles of *Pg* phages in health and disease will also require broader sampling and study of clinical isolates, metagenomes, transcriptomes, and “live” phages from oral samples. Worth remembering in all studies is that just as *Pg* exerts an outsized impact relative to its abundance, *Pg* phages are also exerting their effects from within a milieu of potentially far more abundant phages. Judgments on the presence and activity of *Pg* phages must therefore be made in the context of datasets that are expected to have enough sequencing depth to detect them. Given these constraints, primers targeted to conserved regions of *Pg* phage genomes may provide a useful approach for initial screening studies aiming to broadly assess prevalence and associations of specific phages across states of health and disease.

## Conclusion

This work establishes that phages are important in the ecology of the oral pathogen *Pg* and characterizes representatives of three new candidate viral families including *Pg* phages, the *Alisviridae*, *Nixviridae*, and *Ludisviridae*. The foundational phage sequence datasets and model systems that we establish here add to the rich context of all that is already known about *Pg*, and point to new avenues of inquiry with specific relevance to understanding mechanisms underlying periodontal disease progression. Given the challenges of understanding the complexities of phage-bacteria-human interactions, new model systems in the uniquely well-characterized [1, 98, 99] context of the oral microbiome promise to shed new light on fundamental features of phage impacts on human health and disease broadly.

## Materials and methods

### Bacterial strains and growth conditions

*Porphyromonas gingivalis* isolates Bg4, A7436-C, and HG1691-OLD were shared by Robert E. Schifferle (University at Buffalo, Buffalo, NY) and isolate ATCC 49417 was purchased from the American Type Culture Collection (Manassas, VA). Glycerol stocks of each strain were streaked onto BHI blood agar plates [Brain Heart Infusion (BD Difco Bacto 237500)—37 g/L, sodium bicarbonate (JT Baker 3506-01)—1 g/L, yeast extract (VWR J850)—5 g/L, and L-cysteine (Sigma-Aldrich C7352)—0.5 g/L; then supplemented (post-autoclaving) with hemin (Sigma-Aldrich H9039)—1 mL (5 mg/mL stock concentration), 1,4-dihydroxy-2-naphthoic acid (TCI D2296)—10 mL (0.1 mg/mL stock concentration), and defibrinated sheep blood (Bio Link Inc)—53 mL, adapted from Floyd Dewhirst, Forsyth Institute, MA]. After 5 days (6, for ATCC 49417) of anaerobic incubation at 37 °C in a GasPak jar with an EZ Anaerobe Container System Sachets with Indicator (BD BBL), multiple colonies from each plate were inoculated into two (three, for ATCC 49417) 100-mL volumes, respectively, of pre-reduced, modified ATCC 2722 broth [Tryptic Soy Broth (Soybean-Casein Digest Medium) (BD Bacto 211825)—30 g/L, yeast extract (VWR J850)—5 g/L, and L-cysteine (Sigma-Aldrich C7352)—0.5 g/L; then supplemented (post-autoclaving) with hemin (Sigma-Aldrich H9039)—1 mL (5 mg/mL stock concentration) and 1,4-dihydroxy-2-naphthoic acid (TCI D2296)—10 mL (0.1 mg/mL stock concentration) and anaerobically incubated at 37 °C in a Coy chamber (supplied with 5% CO_2_, 5% H_2_, 90% N_2_) or GasPak jar with an anaerobic sachet (for ATCC 49417). Addition of DHNA was found to be especially beneficial in supporting growth of *Pg* strains, as previously noted [100, 101].

### Bacterial sequencing and genome assembly

At 2 days post-inoculation of Bg4, A7436-C, and HG1691-OLD cultures (1 day for ATCC 49417), 1.5 mL from each of 2 replicate cultures, per strain, was pooled and centrifuged (Beckman Coulter Allegra X-22R Centrifuge with F2402H rotor) at 5000 × *g* (4 °C) for 10 min to pellet the cells (with the exception of A7436-C which required an additional 15 min of centrifugation at 7500 × *g*). After the centrifugation was complete, the supernatants were removed and the pelleted cells were frozen on dry ice and stored at −80 °C. The pellets were extracted and sequenced by the SeqCenter (Pittsburgh, PA) using both Illumina and Nanopore. As reported by SeqCenter: For Illumina sequencing, sample libraries were prepared using the Illumina DNA Prep kit and IDT 10 bp UDI indices and sequenced on an Illumina NextSeq 2000 (2 × 151 bp reads). Demultiplexing, quality control, and adapter trimming were performed with bcl-convert (v3.9.3). For Nanopore sequencing, runs were on a MinION with an R9 pore type (R9.4.1), base calling was done in high accuracy mode, and Guppy v5.0.16 was used. Genome assemblies were then performed in house from sequences of each culture (Bg4 = GCA_029911295.1, A7436-C = GCA_028335105.1, HG1691-OLD = GCA_028335085.1, and ATCC 49417 = GCA_028335125.1; see “Availability of data and materials” statement for link to sequences). In brief, Illumina read quality control was performed by fastp v.0.23.2 [102] [default parameters] (https://github.com/OpenGene/fastp), while Nanopore read quality control was performed by Filtlong v.0.2.1 [default parameters; except minimum length threshold of 1000 and 95% keep percentage of best reads] (https://github.com/rrwick/Filtlong) considering a minimum length threshold of 1000 and keeping 95% of the best reads and Porechop v.0.2.4 [default parameters; except discard reads with middle adaptors] (https://github.com/rrwick/Porechop). Hybrid assemblies with the optimized Illumina and Nanopore reads were produced with Unicycler [103] v.0.5.0 [default parameters] (https://github.com/rrwick/Unicycler). The hybrid assemblies were then polished by Polypolish [104] v.0.5.0 [default parameters] (https://github.com/rrwick/Polypolish) with Illumina read alignments by BWA [105] v.0.7.17 [default parameters] (https://github.com/lh3/bwa) and MaSuRCA [106] v.4.0.9 (using POLCA [107]) [default parameters] (https://github.com/alekseyzimin/masurca).

### Phage sequencing and read mapping

For strains *ATCC 49417, Bg4*, and *HG1691-OLD*, at 19 days post-inoculation (20 days for ATCC 49417), 184 mL (231 mL for ATCC 49417) from each replicate culture (same as those previously described for bacterial sequencing) was pooled, per strain, and filtered using a 0.22-µm filter system (Corning) to remove the cells. Phages were ultracentrifuged (Beckman Coulter Optima XE-90 Ultracentrifuge with SW 32 Ti rotor) at 174,900 × *g* (22 °C) for 1 h (repeated until each culture was completely pelleted by removing the supernatant and refilling the tubes, followed by a rinse centrifugation with SM buffer) in Ultra-Clear centrifuge tubes (Beckman) pre-rinsed with sterile distilled water. After the centrifugation, the supernatant was removed and the pellet was allowed to resuspend overnight (4 days, for ATCC 49417) at 4 °C in enough SM buffer to cover the pellet. The next morning, the pellets were rocked for approximately 2 h at room temperature (~ 22 °C), the resuspended pellet was removed, and each tube washed with approximately 450 µL SM buffer for a total volume of ~ 900 µL for each strain to be used in the phage DNA extraction protocol modified from Jakočiūnė and Moodley 2018 [108]. In brief, the resuspended pellets were each split into two 450-µL samples. The unprotected nucleic acids were removed by adding 50 µL of 10 × Turbo DNase Buffer (Qiagen), 5 µL of 2U/µL Turbo DNase (Qiagen), and 1 µL of 10 mg/mL RNase A (Qiagen), then incubating at 37 °C for 1.5 h without shaking. The nucleases were then denatured by simultaneously adding 20 µl of 0.5 M EDTA and 57 µL of 20 mg/µL Proteinase K (Qiagen) and incubating at 56 °C for 2 h, vortexing every 20 min (an additional 57 µL of Proteinase K was added at 100 (60, for ATCC 49417) min because the sample was still cloudy). The once-protected DNA was then extracted using the DNeasy Blood & Tissue Kit (Qiagen). First, an equal volume of AL Buffer (Qiagen) was added to each sample, these were then vortexed and incubated at 70 °C for 10 min. After incubation, the same volume of 100% ethanol was added and the samples were vortexed. The samples were transferred into DNeasy Mini spin columns (Qiagen) and centrifuged (Beckman Coulter Allegra X-22R Centrifuge with F2402H rotor) at 6000 × *g* (22 °C) for 1 min. This was repeated several times until all of each sample was run through the spin column. Next, 500 mL of Buffer AW1 (Qiagen) was added to the spin columns which were then centrifuged at 6000 × *g* (22 °C) for 1 min. Then, 500 mL of Buffer AW2 (Qiagen) was added to the spin columns which were then centrifuged at 20,000 × gf (22 °C) for 3 min. Lastly, 40 mL of AE Buffer (Qiagen) was added directly onto the spin column membrane and let incubate at room temperature for 1 min prior to centrifugation at 6000 × *g* (22 °C) for 1 min. The collected DNA was then shipped to SeqCenter (Pittsburgh, PA) for Illumina sequencing. The sequenced reads were then mapped back to the bacterial assembly produced from their respective culture with BWA [105] v.0.7.17 [default parameters] and SAMBLASTER [109] v.0.1.26 [default parameters] (https://github.com/GregoryFaust/samblaster). To determine the average coverage per gene, the mapped reads were aligned to a bed file of protein-coding regions with the bedtools [110] v.2.30.0coverage function [default parameters] (https://github.com/arq5x/bedtools2). For strain *A7436-C*, A7436-C cell-free, nuclease-protected DNA was extracted, sequenced, and mapped similarly to that previously described, with the few minor exceptions listed here. First, three 100-mL pre-reduced, modified ATCC 2722 broths were inoculated with multiple colonies from a 5-day-old streak on a BHI blood agar plate. After 17 days of anaerobic incubation in a Coy chamber, the cultures were pooled and left to filter by gravity for 7 days (a time period required by the highly viscous nature of the culture). After the filtration, the filtrate was centrifuged as described above and the pellet was eluted for 4 days at 4 °C. Second, during the nuclease deactivation, no additional Proteinase K was added to the incubation which lasted 1.5 h (due to the sample being clear).

### Additional bacterial and phage sequencing

To highlight reproducibility, we note that an additional ATCC 49417 bacterial culture (derived from the same parent glycerol stock that produced ATCC 49417 assembly GCA_028335125.1) was sequenced and assembled (GCA_028993465.1). Whereas the GCA_028335125.1 assembly yielded a single closed contig, the GCA_028993465.1 yielded an assembly where the prophage region was represented as an independent contig. This difference is interpreted as reflecting differences in relative abundances of extra-chromosomal and integrated versions of one of the prophages between the two cultures. Filtered supernatants of the additional ATCC 49417 cultures were also ultracentrifuged, nuclease-treated, extracted, Illumina sequenced, and the reads mapped onto the GCA_028335125.1 assembly, and found to have similar profiles of nuclease-protected DNA between cultures (Supplementary Fig. 14).

### Electron microscopy of active phages

To determine if active phages are produced from ATCC 49417 lysogens, two different samples were imaged via transmission electron microscopy. The first sample was material from the cell-free ATCC 49417 ultracentrifuged pellet (same as the one described for phage sequencing), prior to nuclease treatment, shown in Fig. 6A, B. The second sample was a separate subculture of ATCC 49417 (from the same stock that also gave rise to the subculture used in the bacterial and phage sequencing), that is shown in Fig. 6C, D. This sample was struck out from a glycerol stock onto a BHI blood agar plate supplemented with 100 μL of a 10:1 dHNA-hemin stock mix and incubated anaerobically at 37 °C. After 5 days of incubation, three 10 μL inoculation loops passed through the tail end of the streak were inoculated into 200 mL of modified ATCC 2722 broth and was incubated anaerobically for 3 days. Both samples were identically prepared on formvar/carbon film 200 mesh copper grids (Ted Pella 01803-F). First, the grids were glow discharged for 5 s to improve their hydrophilicity prior to adding 5 μL of the sample. After 30 s, the sample was drawn off and the grids were rinsed with 5 μL of nuclease-free water (Invitrogen AM9938). The water was then drawn off and the rinse was repeated. Lastly, after the water from the second rinse was drawn off, 5 μL of 1% uranyl acetate in water (Electron Microscopy Sciences 22400–1) was added to the grid, then immediately drawn off to let the grid air dry for 20 min. The grids were imaged at University at Buffalo’s Electron Microscopy Core Lab (Jacobs School of Medicine and Biomedical Sciences, Buffalo, NY) on a Hitachi HT7800 high resolution 120 kV transmission electron microscope with a Gatan Rio 16 CMOS camera capturing 4 k × 4 k pixel images.

### Selection and curation of *Pg* genomes used in bioinformatic analyses

We sought to obtain a comprehensive set of high-quality *Pg* genomes, and ultimately defined two sets for analyses in this work: *Pg*_set88 and *Pg*_set79. We considered three sources of *Pg* genome assemblies for inclusion in this study, as follows. First, we included four of the five genomes sequenced and assembled in house, as described above, excluding GCA_028993465.1 from *Pg*_set88 as a duplicate assembly of ATCC 49417. Second, we considered *Pg* assemblies available in NCBI GenBank (88 assemblies initially). To ensure that our collection of GenBank-derived assemblies was comprehensive and free of mislabeled strains (false *Pgs*), we obtained all assemblies for the genus *Porphyromonas* from GenBank and generated whole genome phylogenies using BacSort [default parameters] (https://github.com/rrwick/Bacsort) with the combined FastANI [111] and Mash [112] approach to generate a distance matrix and tree for visualization with phyloXML [113] and Archaeopteryx (https://www.phylosoft.org/archaeopteryx/). We found that all strains labeled in GenBank as *Pg* were members of a single monophyletic clade containing no unlabeled or mislabeled strains, with *P. gulae* the nearest neighboring clade. Four metagenome-derived assemblies were excluded from *Pg*_set88 on the basis of each of their total sizes being < 2 Mb. Finally, we considered *Pg* assemblies available in GenBank and re-assembled in house (as described above) to explore potential for improved assemblies facilitating detection of phages otherwise split across multiple contigs. In exploratory analyses, we found that re-assemblies did not recover additional prophage regions and thus these were also excluded. Thus, *Pg*_set88 included four genomes sequenced in house and 84 genomes from GenBank. To reduce inflation of feature counts in various analyses resulting from inclusion of near-identical genomes, we assign one assembly as the “primary” assembly in all cases where we have multiple assemblies with the same strain name (A7A1_28, ATCC 33277, ATCC 49417, TDC60, W50, W83), or which are known laboratory-derivatives (e.g., W50/BE1, W50/BR1, A7436C). The set of primary assemblies is identified as *Pg*_set79. Primary assemblies were selected as those with the smaller number contigs, and if the number of contigs was the same then the more recent assembly was selected. In the case of genomes representing derivatives, the parent strain was assigned as the primary assembly. Information on all sequences considered is available in Supplementary Data 1.

### Reference phylogeny, gene annotation, and pangenome analysis of *Pg* genomes

To obtain a reference phylogeny for use throughout our study, we used RiboTree [default parameters] (https://github.com/philarevalo/RiboTree), which considers single-copy ribosomal proteins [114], using *P. gulae* assembly (GCA_000768765.1) as an outgroup. To standardize formatting and functional annotation across all *Pg*_set88 assemblies, we used Bakta [78] [default parameters] (https://github.com/oschwengers/bakta). To define pangenome partitions and regions of genome plasticity (RGPs, runs of predominantly flexible genes), we used PPangGGOLiN [77] [default parameters] (https://github.com/labgem/PPanGGOLiN) with Bakta [78] gene calls.

### Identification of CRISPR-Cas and other defense systems in *Pg* genomes

To identify putative CRISPR-Cas systems, *Pg*_set88 genomes were evaluated using CRISPRCasTyper [55] command line CCTyper [55] v.1.6.4 [default parameters] (https://github.com/Russel88/CRISPRCasTyper) and webserver (https://crisprcastyper.crispr.dk). Full summary data are available in Supplementary Data 5. CCTyper [55] identifies *cas* operons (certain and putative) and CRISPR arrays, annotates each on the basis of repeat and *cas* gene similarity to known systems, and combines this information to identify high-confidence CRISPR-Cas systems. Our summary, data regarding the number of high-confidence CRISPR-Cas systems in the 88 *Pg* genomes excludes cases where the *cas* operon classification was ambiguous and where *cas* genes or CRISPR arrays were identified but could not be readily linked to one another, in some cases likely due to fragmented assemblies. Our analysis of the total number of unique spacers, and the proportion that could be mapped to phages, includes data from all identified CRISPR arrays, including those to which *cas* operons could not be linked, and was performed as follows. All spacers were identified to classes on the basis of the CRISPR-Cas operon assignment by command line CCTyper [55], or by the subsequent classification of the consensus repeat for the array by the CCTyper [55] webserver (accessed 11/13/2022), which offers a more frequently updated repeat classification model. Final standardized sequence orientations of repeats and spacers in all arrays were determined on the basis of the strand of the associated *cas* operon interference module or based on identical (direct or by reverse complement) consensus repeats in systems with assigned directions. In cases of Type I-B, I-C, III-B, and VI-B2 systems, the directionality of the array repeats and spacers was set the same as for the *cas* operon, whereas for Type II-C systems the directionality was set to be the reverse [115]. Using this approach yielded a total of 4016 unique spacer sequences (including those with Ns), 4015 when considering reverse complements. To identify candidate non-CRISPR-Cas defense systems, we also annotated all *Pg*_set88 genomes using the PADLOC [72] webserver (https://padloc.otago.ac.nz/padloc/) with PADLOC-DB v1.4.

### Mapping of CRISPR spacer hits to bacterial and phage genomes

To map CCTyper [55] spacers to bacterial genomes and extracted phages (see below), we used Bowtie [56] v.1.1.1 (default parameters; except allowing for either 0 or 1 mismatch in the target sequence; as shown in Fig. 3) (https://github.com/BenLangmead/bowtie). To also allow evaluation of hits to non-phage sequences, we additionally mapped coverage on a per gene basis in bacterial genomes using the BEDTools [110] annotate function [default parameters]. Exploratory analyses of differences in strand level mapping of spacers from Class 1 systems [where crRNAs bind DNA (Types I-B, I-C, and III-B)], versus Class 2 systems [where crRNAs bind mRNA (Types II-C and VI-B2)] [62, 116], showed no consistent patterns. Both direct and reverse complement mappings were therefore counted for all spacers. We note that even where systems are known to have strand preferences there is generally also representation of the other strand among targets in the spacer array [117], perhaps as a result of trans interactions between different systems [64]. To identify potential matches to phages with conserved protein sequences but divergent nucleotide sequence, translated spacer sequences were mapped to phage proteins using SpacePHARER [57] [default parameters] (https://github.com/soedinglab/spacepharer). To determine whether the *Pg* phages potentially have alternate hosts, we used CRISPROpenDb [65] [default parameters; except allow for 0 mismatches in target sequence] (https://github.com/edzuf/CrisprOpenDB) to map spacer sequences harvested from other species of bacteria to all the bacterial genomes, as well as the extracted phages separately.

### Quantification of prophage and defense system contributions to the *Pg* pangenome

The contribution of prophage genes to the *Pg* pangenome was determined by identifying all bacterial gene families (with prefix mmseq.000837.22272) occurring in prophage regions. The proportion of the pangenome associated with defense was determined by identifying all regions of genome plasticity (RGPs, as defined by PPanGGOLiN [77], and representing runs of predominantly flexible genes) that contained any of the following: any gene families for which any member was identified by CCTyper [55] as part of a CRISPR-Cas systems, any gene families for which any member was identified by PADLOC [72] as a defense system, any gene families not captured by the aforementioned tools but for which any member was annotated with functions containing defense-function related keywords (e.g., cas, CRISPR, restriction, abortive infection, Abi, death on curing, addiction, toxin/antitoxin). One gene family identified as defense related but annotated as a transposon was excluded (mmseq.000837.22272.2484). Any RGPs containing any predicted defense-related proteins were considered as potential defense islands or elements, and all non-core gene families in all of these RGPs were counted toward the estimate of total gene families represented by defense islands or elements.

### Identification of prophages in *Pg* genomes

To identify prophages in *Pg*_set88 genomes, we combined multiple complementary approaches and used Geneious Prime versions 2023.0.1 and 2022.2.2 (Biomatters Ltd.) to view all results together and manually curate prophage boundaries. As initial exploratory investigations revealed that some prophage regions were fragmented, our analysis of the *Pg* genomes included a set of “fusion contigs” generated by manual targeted curation to identify contigs encoding genes for which there was evidence of terminal overlap. Fusion contigs were generated for 3 strains (as noted in Supplementary Data 1), with these contigs renamed with terminal “9”s to indicate their having been updated from their original assemblies (e.g., JAEMBP01999999.1 fusion contig from contigs JAEMBP010000058.1 and JAEMBP010000009.1). All *Pg*_set88 genomes, including updated fusion contigs, were then searched for predicted prophage regions using CenoteTaker2 [81] [default parameters] (https://github.com/mtisza1/Cenote-Taker2), VIBRANT [118] [default parameters] (https://github.com/AnantharamanLab/VIBRANT), PhageBoost [119] [default parameters] (https://github.com/ku-cbd/PhageBoost), VirSorter2 [120] [default parameters] (https://github.com/jiarong/VirSorter2) post-processed with CheckV [121] [default parameters] (https://bitbucket.org/berkeleylab/CheckV/src), and Inovirus detector [122] [default parameters] (https://github.com/simroux/Inovirus). To facilitate determination of nucleotide-level boundaries of phage regions, we used an all-by-all BLAST of all *Pg*_set88 genomes. As described above, to facilitate detection of regions targeted by CRISPR spacers, we identified all CRISPR array spacers in *Pg*_set88 and mapped these back to all *Pg*_set88 genomes using Bowtie [56], and we also identified all sites targeted by CRISPR spacers encoded in other bacterial species using CRISPROpenDB [65]. All contigs with predicted prophage regions were then imported into Geneious and evaluated together with tracks showing pangenome partition information for all bacterial genes, all-by-all BLAST results, and CCTyper [55] and CRISPROpenDB [65] spacer mappings. Repeats surrounding candidate regions were next identified using the Geneious Repeat Finder v1.0.1, and final boundaries defined based on identification of bounding repeats proximal to conserved BLAST hit edges (identifying regions commonly showing gaps in *Pg* genomes) and corresponding to regions identified as flexible pangenome partitions. This approach readily revealed boundaries for tRNA-inserting phages, which generally had bounding repeats of ≥ 13 bp (with one repeat being part of the phage genome); however, for the transposable phages, additional curation was needed and included extraction and alignment of candidate regions to identify conserved termini and short 4 bp bounding repeats (both outside the boundaries of the phage genome).

### Prediction of *Pg* phage genes

Exploratory analyses revealed that predicted open reading frames in prophage regions were inconsistently identified both by Bakta [78] in the original bacterial genome annotations, and by Prodigal [123] run separately on only the extracted prophage regions. Therefore, all prophage regions were re-analyzed with CenoteTaker2 [81], which provides excellent functional annotation of open reading frames predicted using PHANOTATE [124] [default parameters] (https://github.com/deprekate/PHANOTATE), a gene caller optimized for phage genes. All protein-coding genes predicted PHANOTATE [124] were clustered using the mmseqs2 [125] easy-cluster function (https://github.com/soedinglab/MMseqs2) and thus phage regions have two sets of protein clusters in our study, those derived from the original Bakta [78] gene calls in the bacterial genomes (identified with the prefix mmseq.000837.22272), and those derived from PHANOTATE [124] gene calls on the extracted phage genomes (identified with the prefix mmseq.010239.22272).

### Annotation of *Pg* phage genes

All phage gene annotations were performed on PHANOTATE [124] derived gene calls as described above. Phage proteins were annotated for predicted function by comparison to the PHROGS [126] v4 database (https://phrogs.lmge.uca.fr/index.php) using 3 iterations of HHblits [127] [default parameters] (https://github.com/soedinglab/hh-suite) and allowing automatic assignment to top hit annotations and categories with a bitscore of > 30, where not superseded by another annotation. Additional annotations included those provided by CenoteTaker2 [81], Bakta [78] using PHANOTATE [124] gene calls as input, eggNOG-mapper [128, 129] (http://eggnog-mapper.embl.de/), Batch CD-Search [130–134] (https://www.ncbi.nlm.nih.gov/Structure/bwrpsb/bwrpsb.cgi), Phyre2 [135] (http://www.sbg.bio.ic.ac.uk/phyre2), HHpred through the MPI Bioinformatics Toolkit [136], SignalP6.0 [137] (https://services.healthtech.dtu.dk/service.php?SignalP-6.0, using “Fast” model option for initial run and “Slow” model option for refining cleavage sites of candidates identified in initial run), and jackhmmer [138] (https://www.ebi.ac.uk/Tools/hmmer/search/jackhmmer). Candidate anti-CRISPR (acr) genes were predicted using two approaches. First, direct annotation of phage protein-coding genes on the PaCRISPR [59] webserver (https://pacrispr.erc.monash.edu/index.jsp). Second, proteins in the DeepAcr database were mapped to PHROG gene families with mmseqs2 [125] [default parameters], and any *Pg* phage gene that was identified as also mapping to the same PHROG was annotated as an acr. Only phage genes identified through both approaches were ultimately annotated as candidate acrs and colored accordingly in the Fig. 2 phage genome diagrams. Select candidate spanins were identified using tools available on the Center for Phage Technology Galaxy Server [139] (https://cpt.tamu.edu/galaxy-pub) (run errors resulted in lack of even annotation across all phage genes) and often showed frameshifts from open reading frames identified by PHANOTATE [124]; in addition, information on lipoprotein signal peptides and proximity to other lysis cassette genes such as the endolysin and holin were also considered. Phage morphotypes and head-neck-tail components were predicted using VIRFAM [41] (http://biodev.cea.fr/virfam/). Except in the case of annotation of anti-crispr proteins, in cases where only a single representative of a protein cluster was annotated (e.g., with Phyre2 [135]), annotations from any member were propagated to all other members of the protein cluster and annotations overall were harmonized within protein clusters. All annotations of phage protein-coding genes are available in Supplementary Data 4.

### Exploratory mapping of healthy and periodontal disease metagenomes to *Pg* phages

Illumina reads from publicly available metagenomic samples from a study [29] of six healthy individuals and seven with periodontitis (≥ 6 mm pockets with bleeding on probing) were downloaded from the Human Oral Microbiome Database [140] (https://homd.org/ftp/publication_data/20130522/). The reads from each patient were mapped to each of the 35 *Pg* reference phage genomes using Geneious Prime v.2022.2.2 (Biomatters Ltd.) with the Geneious mapper at default settings, with the exception of mapping multiple best matches to all locations (such that reads mapping to multiple phages would be represented in coverage mappings from each). The sample from periodontitis donor 3 had the most reads (255,891/12,263,433) map to any particular phage, in this case phage012 (as shown in Supplementary Fig. 2).

### Analysis and visualization of phage genome relatedness

To determine whether any of the phages identified in this work were related to known phages or other phages in bacterial genomes or metagenomes, a stepwise approach was used. All *Pg* prophage genomes were first clustered with all phages (4,912) in the ViPTree [31] v3.5 Virus-Host DB [32] reference set, based on RefSeq release 217, using vConTACT2 [30] v0.9.19 in the Cyverse Discovery Environment [141]. To also explore the relationships of *Pg* prophages to uncultivated phages, we clustered the *Pg* phage genomes with all Uncultivated Viral Genomes (UViGs) in IMG/VRv4 [39] predicted to have hosts in the *Porphyromonadaceae* (1138), using vConTACT2 [30]. Results of these two analyses were visualized as networks using Cytoscape [142] v3.9.1 with the Prefuse Force Directed Layout [Edge Weight Settings: Heuristic, 0 min edge weight, 1.79769E308 max edge weight, 0.5 default edge weight, 1000 iterations, 8E-7 spring coefficient, 60 default spring length, 3 default node mass]. We note that the clusters represented in the Cytoscape [142] visualizations may encompass multiple different viral clusters (VCs) as defined by vConTACT2, and it was the latter more closely related members of VCs that we included from each dataset in subsequent analyses. To identify additional related phages, we whole genome comparisons of *Pg* phages identified in this study with ViPTree [31] references to generate a hierarchically clustered view of neighbors in the reference dataset. To resolve family-level units, we then considered together: all full-length *Pg* phages identified in this study, all phage references in the VipTree [31] clade that contained all *Pg* phages, phage isolates and metagenomic sequences identified as *Winoviridae* in NCBI and the related publication [36], phages related to but outside the *Winoviridae* [36], and uncultivated and metagenomic phages identified as clustering in vConTACT2 VCs with the *Pg* phages. We evaluated relationships among this set of 82 phages using VIRCLUST [33] and VICTOR [28]. VIRCLUST [33] resolved the *Pg* phages into three distinct family-level units on the basis of shared core Protein Super Clusters, and this was corroborated by VICTOR [28] subfamily taxon predictions (based on amino acids and d6 distance formula), which have been shown to offer the best correspondence to dsDNA phage family-level units as currently recognized by the International Committee on the Taxonomy of Viruses [33, 36]. These analyses support the three *Pg* phage clusters as representing three candidate family-level units, with the transposable phage candidate family containing the currently unclassified *Bacteroides dorei* Hankyphage p00 [35], the tRNA-ser candidate family containing the currently unclassified *Riemerella anatipestifer* phage RAP44 [37, 38], and the tRNA-pro candidate family containing no previously characterized phages. To resolve genus- and species-level groups, we used VIRIDIC [34] nucleotide-based whole genome distances among the *Pg* phages (95 and 70% nucleotide identity thresholds, respectively).

### Bioinformatic analyses

Unless otherwise specified above, bioinformatic analyses were conducted on the Center for Computational Research at University at Buffalo [143] high performance compute cluster﻿ using Miniconda (https://docs.conda.io/en/latest/miniconda.html), conda environments (https://docs.conda.io/en/latest/) installed from the Anaconda Package Repository (https://anaconda.org/anaconda/repo), and in house Unix shell script wrappers.

### Supplementary Information

**Additional file 1: Supplementary Figure 1.** Integration of complementary bioinformatic approaches unveiled numerous *Porphyromonas gingivalis* prophages. Example view from Geneious bioinformatic software highlighting numerous analyses used in manually curating *Pg* prophages. Bacterial contig CP024591 (KCOM 2802) was searched with five prophage predicting tools (VirSorter2 [120] with CheckV [121], Cenote-Taker2 [81], PhageBoost [119], VIBRANT [118], and Inovirus Detector [122]); hits indicated in yellow bars. Annotations performed by Cenote-Taker2 [81] aided in determining the validity of the phage predictions through sensitive detection of major capsid proteins (marked by white stars). Pangenome partitions, predicted by PPanGGOLiN [77], designate “flexible” protein-coding genes (light blue and light green block arrows), as compared to those that are “core” (orange block arrows); direct repeats were also identified as an indicator of insertion (those used by the phage marked by white triangles). Matches of CRISPR spacers (100% identity) found from *Pg* strains (shown as blue hash marks; identified by CCTyper [55]) and strains of other species (shown as dark blue hash marks; mapped from CRISPROpenDB [65]) elucidate regions targeted by intra- and interpopulation CRISPR-Cas systems, respectively. All-by-all intrapopulation BLAST used to compare each *Pg* genome against all other *Pg* genomes shows areas that lack conservation; hits indicated by gray bars. The final manually curated prophage region (phage026 with functional annotations), inserted into a tRNA-pro gene (pink block arrow), is defined taking into account all analyses.

**Additional file 2: Supplementary Figure 2.** Transposable *Porphyromonas gingivalis *phages are enriched in metagenomic reads from a periodontitis patient. Coverage (dark gray plot) of a transposable phage (phage012a_381OKJ) by metagenomic sequences sampled from the oral cavity of a periodontitis patient. Reads mapped to the entire phage genome, with maximum 1,195x coverage (indicated by the scale on the left, middle hash mark notes mean coverage). A preliminary search with these reads sequenced from the same patient showed lower coverage mappings to other transposable *Pg* phages. Colored block arrows represent phage functional gene groups (major capsid protein marked with star) predicted by Cenote-Taker2 [81].

**Additional file 3: Supplementary Figure 3.** Identification of relatives of *Porphyromonas gingivalis* phages among reference phages, on the basis of vConTACT2 proteome sequence similarity. Network representation of vConTACT2 [30] whole proteome similarity among all *Pg* phages and 4,912 dsDNA Prokaryote-infecting viruses in the ViPTree [31] v3.5 Virus-Host DB [32] reference set, based on RefSeq release 217. Nodes represent viral genomes and are colored based on family-level classification, determined per ViPTree [31] and Inphared [145] (1May2023_itol_family_annotations), with colors defined per those assigned in the latter. Network clusters containing *Pg* phages identified in this study are highlighted with colored boxes; note that vConTACT2 [30] defines cohesive Viral Clusters (VCs) that may contain only subsets of nodes appearing together in the same network cluster (see Supplementary Data 3).

**Additional file 4: Supplementary Figure 4.** Identification of nearest-neighbors of *Porphyromonas gingivalis *phages among reference phages, on the basis of ViPTree tBLASTx-based intergenomic distances. Placement of *Pg* phages among most sequence-similar reference phages in the 4,912 dsDNA Prokaryote-infecting viruses in the ViPTree [31] v3.5 Virus-Host DB [32] reference set, based on genome-wide tBLASTx-based sequence similarities. *Pg* phages are highlighted with labels colored corresponding to their insertion group type and completeness, reference phages in named families are indicated with boxes in shades of grey adjacent to their names (I), and reference phages infecting in the *Bacteroidetes* are indicated with a brown box adjacent to their names (II). All sequences included in this clade are reported in Supplementary Data 3.

**Additional file 5: Supplementary Figure 5.** Resolution of *Porphyromonas gingivalis *phages to three family level units, on the basis of VirClust Protein Super Cluster (PSC)-based intergenomic distances. The set of 82 phages included in this analysis was comprised of: all full-length *Pg* phages identified in this study (this excluded duplicates identified in alternate assemblies of the same *Pg* strain); all *Porphyromonadaceae* UViGs in IMG/VRv4 [39] assigned to the same vConTACT2 [30] Viral Cluster with the *Pg* phages, but not including those representing redundant geNomad [40] versions of the *Pg* phages; all reference phages identified in the ViPTree [31] placement tree as occurring within the same clade containing all* Pg* phages; all representatives of the closely-related viral family *Winoviridae* [36] identified in GenBank and the publication describing this group, as well as phages identified in the aforementioned publication as potentially related to the *Winoviridae* but lying outside the family (e.g. *Bacteroides* phage p00 and *Cellulophaga* phage phi46); all sequence accessions are reported in Supplementary Data 3. The VirClust [33] tree on the left reflects hierarchical clustering based on whole genome protein supercluster similarity; the silhouette width measures relatedness of a virus to other viruses within its own viral genome cluster (VGC) and to viruses outside of its VGC, with -1 indicating greatest similarity to viruses in other VGCs and 1 indicating greatest similarity to viruses within the same VGC (none <0, only range from 0 to 1 shown); the matrix represents all protein super clusters (PSCs) identified in the entire dataset (columns), with the number of PSCs per genome indicated by cell color. *Pg* phages are highlighted with leaf labels colored corresponding to insertion group type and completeness, clades identified as distinct family-level clusters by VirClust [33] are highlighted with dashed outlines, named and proposed families of phages are indicated in italics and underlined italics, respectively.

**Additional file 6: Supplementary Figure 6.** Resolution of *Porphyromonas gingivalis *phages to three family level units, on the basis of VICTOR whole proteome intergenomic distances. The set of 82 phages included in this analysis was comprised of: all full-length *Pg* phages identified in this study (this excluded duplicates identified in alternate assemblies of the same *Pg* strain); all *Porphyromonadaceae* UViGs in IMG/VRv4 [39] assigned to the same vConTACT2 [30] Viral Cluster with the *Pg* phages, but not including those representing redundant geNomad [40] versions of the *Pg* phages; all reference phages identified in the ViPTree placement tree as occurring within the same clade containing all* Pg* phages; all representatives of the closely-related viral family *Winoviridae* [36] identified in GenBank and the publication describing this group, as well as phages identified in the aforementioned publication as potentially related to the *Winoviridae* but lying outside the family (e.g. *Bacteroides* phage p00 and *Cellulophaga* phage phi46); all sequence accessions are reported in Supplementary Data 3. The tree on the left reflects whole proteome similarity based on VICTOR [28] d6 (recommended for amino acid datasets) formula whole proteome distances, with branch supports based on 100 pseudo-bootstrap replicates; *Pg* phages are highlighted with leaf labels colored corresponding to insertion group type and completeness; clades identified as distinct subfamily-level clusters by VICTOR [28] (best corresponding to currently accepted thresholds for ICTV viral families) are indicated with colored boxes and highlighted with dashed outlines for named and proposed families of phages, indicated in italics and with underlines, respectively.

**Additional file 7: Supplementary Figure 7.** Genome diagrams of *Porphyromonas gingivalis* phages show conservation of protein clusters. *Pg* phage phylogeny (30 full, 5 partial; names of full length phages are in saturated colors and partial phages are in lighter shades; “b” suffix indicates version of an “a” phage found in a different assembly of the *Pg *strain; midpoint-rooted tree based on whole genome nucleotide BLAST distance scaled by VICTOR [28] d0 formula, recommended for nucleic acid datasets) and genome diagram (generated using Clinker [42]) as shown in Fig. 2, with the exception that the predicted protein-coding genes (depicted as block arrows) are colored based on sequence similarity. Thus, highlighting the conservation of protein clusters and ordering among related *Pg* phages. Candidate genus- and species-level clusters are shown for full-length phages in the yellow bars. Three higher-order clades of phages defined by distinct insertion sites in host genomes (by full-length phages only) are highlighted by coloring of phage names (orange: transposition-based insertion; purple: tRNA-ser; green: tRNA-pro). White stars mark phage genome ends defined by contig ends, circles mark phage genomes identified in this work by joining contigs with overlapping termini, the dotted line in the middle of phage033a highlights that this phage was identified at the two termini of a bacterial contig assembly and is missing genes potentially due to an incomplete assembly.

**Additional file 8: Supplementary Figure 8.** Identification of relatives of *Porphyromonas gingivalis *phages among phages in genomic- and metagenomic-datasets in IMG/VRv4, and predicted to infect hosts in the *Porphyromonadaceae*. Network representation of vConTACT2 [30] whole proteome similarity among all *Pg* phages and 1,138 uncultivated viral genomes (UViGs) in IMG/VRv4 [39] known or predicted to infect hosts in the *Porphyromonadaceae*. Nodes represent viral genomes and are colored based on known or predicted host species, with triangles identifying *Pg* phages identified in this study. Network clusters containing *Pg* phages identified in this study are highlighted with colored boxes; note that vConTACT2 [30] defines cohesive Viral Clusters (VCs) that may contain only subsets of nodes appearing together in the same network cluster (see Supplementary Data 3).

**Additional file 9: Supplementary Figure 9.** Transposable *Porphyromonas gingivalis *phages identified in this study share synteny with UViGs. All uncultivated viral genomes (UViGs) reported in IMG/VRv4 [39] as predicted to infect *Porphyromonadaceae* were clustered with *Pg* phages on the basis of shared proteins using vConTACT2 [30]. All UViGs assigned to the same vConTACT2 [30] Viral Cluster as the transposable *Pg* phages are shown here and were aligned with the *Pg* phages using Clinker [42] v0.0.27, with ordering based on *Pg* phage only tree shown in Fig. 2 and placement of UViGs assisted by a whole genome distance tree generated using the VICTOR [28] d4 distance formula (recommended for datasets with numerous different length sequences) with nucleic acid input. In addition to the ten transposable *Pg* phages identified in this study, three UViGs are shown: two IMG/VRv4 [39] geNomad [40] pipeline versions of the same prophages (represented by double-headed arrows) and one prophage predicted in a *Porphyromonas gulae* genome. Also shown are the known or predicted phage host group (I), environmental source (II), and sequence source (III). The *P. gulae* UViG is predicted to represent a distinct species-level group within the same genus-level group as the *Pg* phages, as determined based on whole-genome nucleotide similarity with VIRIDIC [34].

**Additional file 10: Supplementary Figure 10. ***Porphyromonas gingivalis *phages with tRNA-serine gene insertion sites identified in this studying share synteny with UViGs. All uncultivated viral genomes (UViGs) reported in IMG/VRv4 [39] as predicted to infect *Porphyromonadaceae* were clustered with *Pg* phages on the basis of shared proteins using vConTACT2 [30]. All UViGs assigned to the same vConTACT2 [30] Viral Cluster as the tRNA-ser inserting *Pg* phages are shown here and were aligned with the *Pg* phages using Clinker [42] v0.0.27, with ordering based on *Pg* phage only tree shown in Fig. 2 and placement of UViGs assisted by a whole genome distance tree generated using the VICTOR [28] d4 distance formula (recommended for datasets with numerous different length sequences) with nucleic acid input. In addition to the five (partial- and full-length) tRNA-ser *Pg* phages identified in this study, 16 UViGs are shown: four IMG/VRv4 [39] geNomad  [40] pipeline versions of the same prophages (represented by double-headed arrows) and 12 UViGs predicted from oral and intestinal metagenomes. Also shown are the known or predicted phage host group (I), environmental source (II), and sequence source (III). The *Pg *phages represent a distinct genus-level group from the UViGs identified in the metagenomic datasets, as determined based on whole-genome nucleotide similarity with VIRIDIC [34].

**Additional file 11: Supplementary Figure 11. ***Porphyromonas gingivalis* phages with tRNA-proline gene insertion sites identified in this studying share synteny with UViGs. All uncultivated viral genomes (UViGs) reported in IMG/VRv4  [39] as predicted to infect *Porphyromonadaceae* were clustered with *Pg* phages on the basis of shared proteins using vConTACT2 [30]. All UViGs assigned to the same vConTACT2 [30] Viral Cluster as the tRNA-pro inserting *Pg* phages are shown here and were aligned with the *Pg* phages using Clinker [42] v0.0.27, with ordering based on *Pg* phage only tree shown in Fig. 2 and placement of UViGs assisted by a whole genome distance tree generated using the VICTOR [28] d4 distance formula (recommended for datasets with numerous different length sequences) with nucleic acid input. In addition to the 18 (partial- and full-length) tRNA-pro *Pg* phages identified in this study, 13 UViGs are shown: 11 IMG/VRv4 [39] geNomad [40] pipeline versions of the same prophages (represented by double-headed arrows), one predicted *Porphyromonas gulae* prophage, and one UViG from an intestinal metagenome. Also shown are the known or predicted phage host group (I), environmental source (II), and sequence source (III). Whereas the intestinal UViG is predicted to belong to the same genus as some of the* Pg* phages, the *P. gulae* prophage represents a distinct genus-level group, as determined based on whole-genome nucleotide similarity with VIRIDIC [34].

**Additional file 12: Supplementary Figure 12.** Consensus sequences for repeats within individual *Porphyromonas gingivalis* CRISPR-Cas arrays show intermingling within some systems and sequence diversity within others. Type I-B and I-C arrays share common consensus repeats (A), whereas Type II-C (B) and Type III-B (C) arrays have distinct consensus repeats. Notably, for the Type VI-B arrays, there are two distinct groups of conserved repeats, one of these groups is associated with Type VI-B arrays that are part of the *Pg* core genome (D), whereas the other group (E) is associated with flexible Type VI-B systems. Consensus repeats shown are those from *Pg*_set79, in standardized orientation, and excluding repeats for which no CRISPR-Cas system type could be predicted (underlying data available in Supplementary Data 5).

**Additional file 13: Supplementary Figure 13.** Pangenomic partitioning of all 88 *Porphyromonas gingivalis* strains reveals an abundance of flexible genes. Plot indicates the number of gene families occurring in each of a given number of *Pg* genomes, from gene families that occur in only one genome to gene families that are found in all 88 (as predicted by PPanGGOLiN  [77] using clustered proteins for *Pg*_set88). Light blue and green bars represent counts of gene families with “cloud” and “shell” designations by PPanGGOLiN  [77] (combined and referred to in the text as making up the “flexible” pangenome), respectively, while orange bars represent “persistent” designations (referred to in the text as making up the “core” pangenome).

**Additional file 14: Supplementary Table 1.** Summary information describing differences between sets of *Pg* genomes used in this study, with *Pg*_set_88 including all genomes and *Pg*_set_79 including only representatives of each strain to eliminate inflation of feature counts in various analyses resulting from inclusion of near-identical genomes.

**Additional file 15: Supplementary Figure 14.** Read mapping of filtered, nuclease-treated supernatants reveals the presence of protected, extracellular DNA in *Porphyromonas gingivalis* cultures. Mapping of Illumina sequencing reads from DNA extracted from cell-free, nuclease-treated, ultracentrifuged pellets of supernatants of ATCC 49417 *Pg* cultures. Bottom mapping represents reads from “Experiment 1”, which used supernatants from a 19-day old culture; an aliquot of the same culture at a younger age was used to obtain cell pellets from which assembly GCA_028993465 was produced. Top mapping represents reads from “Experiment 2”, which used supernatants from a 20-day old culture; an aliquot of the same culture at a younger age was used to obtain cell pellets from which assembly GCA_028335125 was produced. Both cultures were struck from glycerols originally derived from the same parent glycerol. Reads from both experiments were mapped onto the closed GCA_028335125 assembly and show coverage spikes along the genome (dark gray plots). Regions with greater than 500x coverage are marked by dark gray bars along the length of the reference, regions encoding phage005 and phage019 and marked by purple and green bars, respectively. Select additional peaks of high coverage are also shown, with clusters of elevated coverage named for the gene of highest coverage within the cluster or, where the peak gene is a hypothetical, with the name indicating another gene of known function nearby in the cluster. Mean coverage data for each protein-coding gene in the reference assembly is provided in Supplementary Data 11.

**Additional file 16: Supplementary Data 01.** Overview of bacterial and phage genome information.

**Additional file 17: Supplementary Data 02.** Virulence.

**Additional file 18: Supplementary Data 03.** vConTACT2, VirClust, and VICTOR analyses.

**Additional file 19: Supplementary Data 04.** Phage protein coding gene annotations.

**Additional file 20: Supplementary Data 05.** CRISPR-Cas system annotations.

**Additional file 21: Supplementary Data 06.** CCtyper CRISPR spacer hits to phages.

**Additional file 22: Supplementary Data 07.** SpacePHARER CRISPR spacer hits to phages.

**Additional file 23: Supplementary Data 08.** CRISPROpenDB CRISPR spacer hits to phages.

**Additional file 24: Supplementary Data 09.** Bacterial protein coding gene annotations.

**Additional file 25: Supplementary Data 10.** Transposases.

**Additional file 26: Supplementary Data 11.** Mapping of cell-free nuclease-protected DNA to *Pg* ATCC 49417.

## Data Availability

All *Pg* genomes sequenced for this work have been deposited to NCBI GenBank BioProject PRJNA874424 (https://www.ncbi.nlm.nih.gov/bioproject/PRJNA874424) with BioSample accession numbers SAMN30559729-SAMN30559733. All other data not already included in Supplementary Data files or described here, as well as any wrapper shell scripts used to run described publicly available bioinformatic tools, are available upon request from the authors. All sequence files and outputs from clustering tools used for family-level taxonomic classification are available at: https://zenodo.org/record/7489346. We encourage those who are interested to consult the readme in Zenodo regarding different versions of assembly identifiers and locus tag names between NCBI, Zenodo, and the Supplementary Data Files for strains sequenced in this study, a consequence of PGAP [144] re-annotation of these assemblies upon submission to NCBI.
