# Correction: Phages are unrecognized players in the ecology of the oral pathogen *Porphyromonas gingivalis*

**DOI:** 10.1186/s40168-024-01880-3

**Published:** 2024-08-05

**Authors:** Cole B. Matrishin, Elaine M. Haase, Floyd E. Dewhirst, Jessica L. Mark Welch, Fabiola Miranda‑Sanchez, Tsute Chen, Donald C. MacFarland, Kathryn M. Kauffman

**Affiliations:** 1 Department of Oral Biology, School of Dental Medicine, The University at Buffalo, Buffalo, NY USA; 2 Department of Microbiology, The Forsyth Institute, Cambridge, MA USA; 3 Department of Oral Medicine, Infection and Immunity, Harvard School of Dental Medicine, Boston, MA USA; 4 Department of Pathology and Anatomical Sciences, Jacobs School of Medicine, The University at Buffalo, Buffalo, NY USA

**Correction** **:** **Microbiome 11, 161 (2023)**

**https://doi.org/10.1186/s40168-023-01607-w**

Following publication of the original article [1], the author requested that the phage NCBI GenBank accession numbers should be added to “Additional file 16: Supplemental Data 01. This additional information will allow readers to easily find these phage sequences in the NCBI database.

The updated Additional file 16 is included in this erratum and the original article has been updated.

### Supplementary Information

Supplementary Material 1.
