# Supplementary figures and images for: Phages are unrecognized players in the ecology of the oral pathogen Porphyromonas gingivalis
Source: Microbiome. 2023 Jul 25;11:161. doi: 10.1186/s40168-023-01607-w (PMC10367356; doi:10.1186/s40168-023-01607-w)

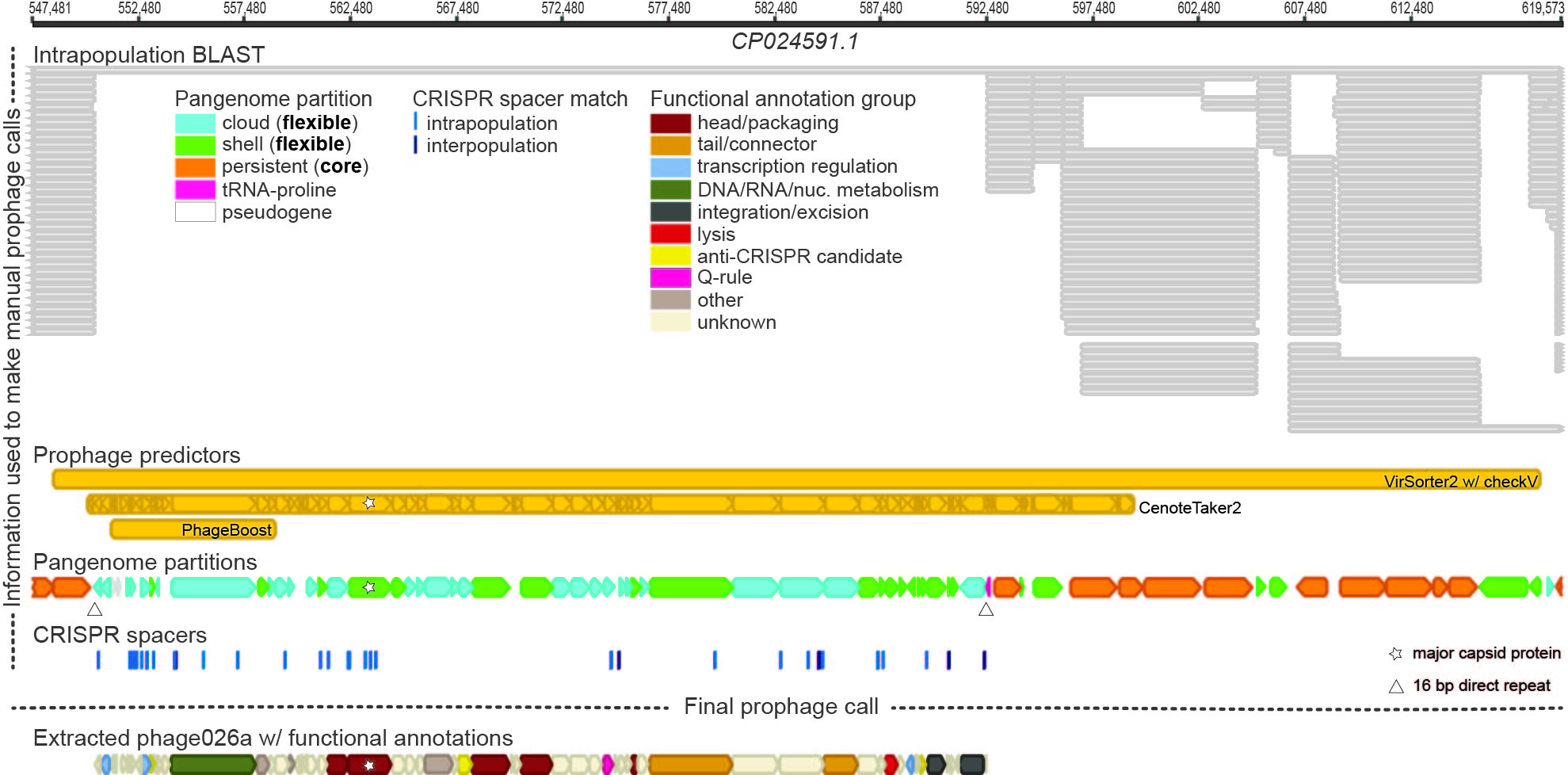

Supplement: Supplementary file 2 — Additional file 1: Supplementary Figure 1. Integration of complementary bioinformatic approaches unveiled numerous Porphyromonas gingivalis prophages. Example view from Geneious bioinformatic software highlighting numerous analyses used in manually curating Pg prophages. Bacterial contig CP024591 (KCOM 2802) was searched with five prophage predicting tools (VirSorter2 [120] with CheckV [121], Cenote-Taker2 [81], PhageBoost [119], VIBRANT [118], and Inovirus Detector [122]); hits indicated in yellow bars. Annotations performed by Cenote-Taker2 [81] aided in determining the validity of the phage predictions through sensitive detection of major capsid proteins (marked by white stars). Pangenome partitions, predicted by PPanGGOLiN [77], designate “flexible” protein-coding genes (light blue and light green block arrows), as compared to those that are “core” (orange block arrows); direct repeats were also identified as an indicator of insertion (those used by the phage marked by white triangles). Matches of CRISPR spacers (100% identity) found from Pg strains (shown as blue hash marks; identified by CCTyper [55]) and strains of other species (shown as dark blue hash marks; mapped from CRISPROpenDB [65]) elucidate regions targeted by intra- and interpopulation CRISPR-Cas systems, respectively. All-by-all intrapopulation BLAST used to compare each Pg genome against all other Pg genomes shows areas that lack conservation; hits indicated by gray bars. The final manually curated prophage region (phage026 with functional annotations), inserted into a tRNA-pro gene (pink block arrow), is defined taking into account all analyses. [file 40168_2023_1607_MOESM1_ESM.jpg]

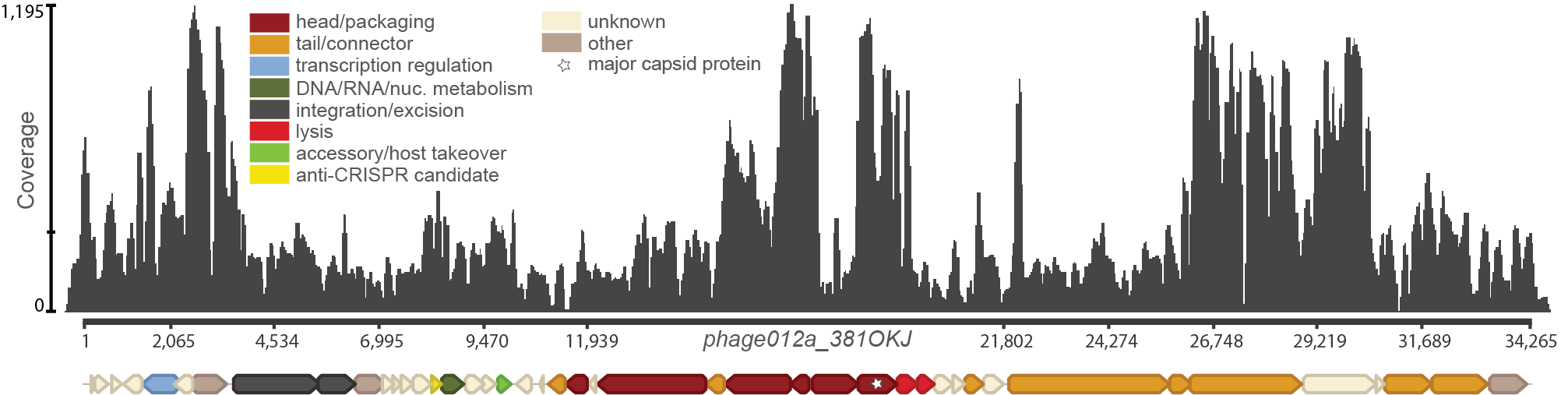

Supplement: Supplementary file 3 — Additional file 2: Supplementary Figure 2. Transposable Porphyromonas gingivalis phages are enriched in metagenomic reads from a periodontitis patient. Coverage (dark gray plot) of a transposable phage (phage012a_381OKJ) by metagenomic sequences sampled from the oral cavity of a periodontitis patient. Reads mapped to the entire phage genome, with maximum 1,195x coverage (indicated by the scale on the left, middle hash mark notes mean coverage). A preliminary search with these reads sequenced from the same patient showed lower coverage mappings to other transposable Pg phages. Colored block arrows represent phage functional gene groups (major capsid protein marked with star) predicted by Cenote-Taker2 [81]. [file 40168_2023_1607_MOESM2_ESM.png]

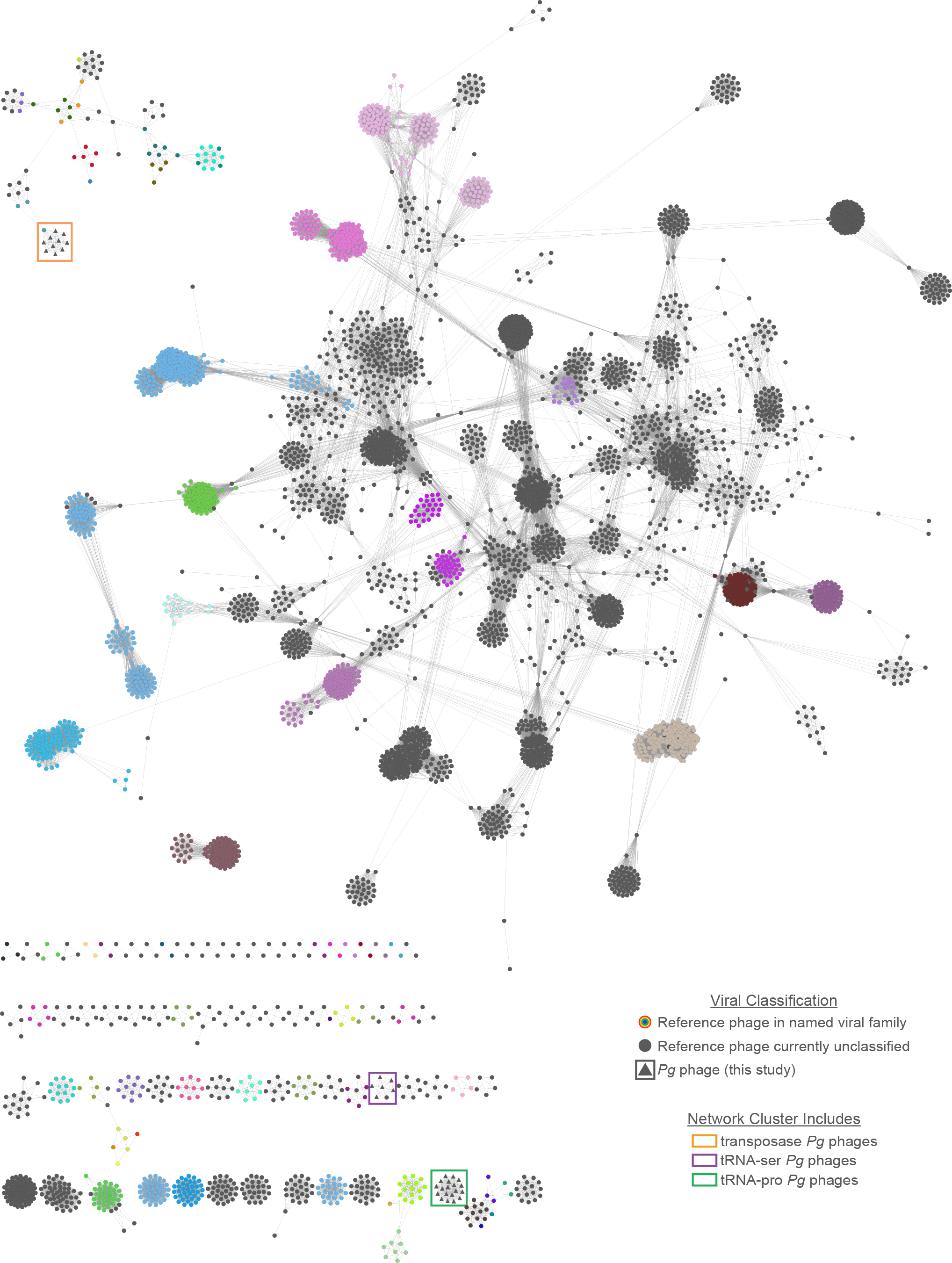

Supplement: Supplementary file 4 — Additional file 3: Supplementary Figure 3. Identification of relatives of Porphyromonas gingivalis phages among reference phages, on the basis of vConTACT2 proteome sequence similarity. Network representation of vConTACT2 [30] whole proteome similarity among all Pg phages and 4,912 dsDNA Prokaryote-infecting viruses in the ViPTree [31] v3.5 Virus-Host DB [32] reference set, based on RefSeq release 217. Nodes represent viral genomes and are colored based on family-level classification, determined per ViPTree [31] and Inphared [145] (1May2023_itol_family_annotations), with colors defined per those assigned in the latter. Network clusters containing Pg phages identified in this study are highlighted with colored boxes; note that vConTACT2 [30] defines cohesive Viral Clusters (VCs) that may contain only subsets of nodes appearing together in the same network cluster (see Supplementary Data 3). [file 40168_2023_1607_MOESM3_ESM.png]

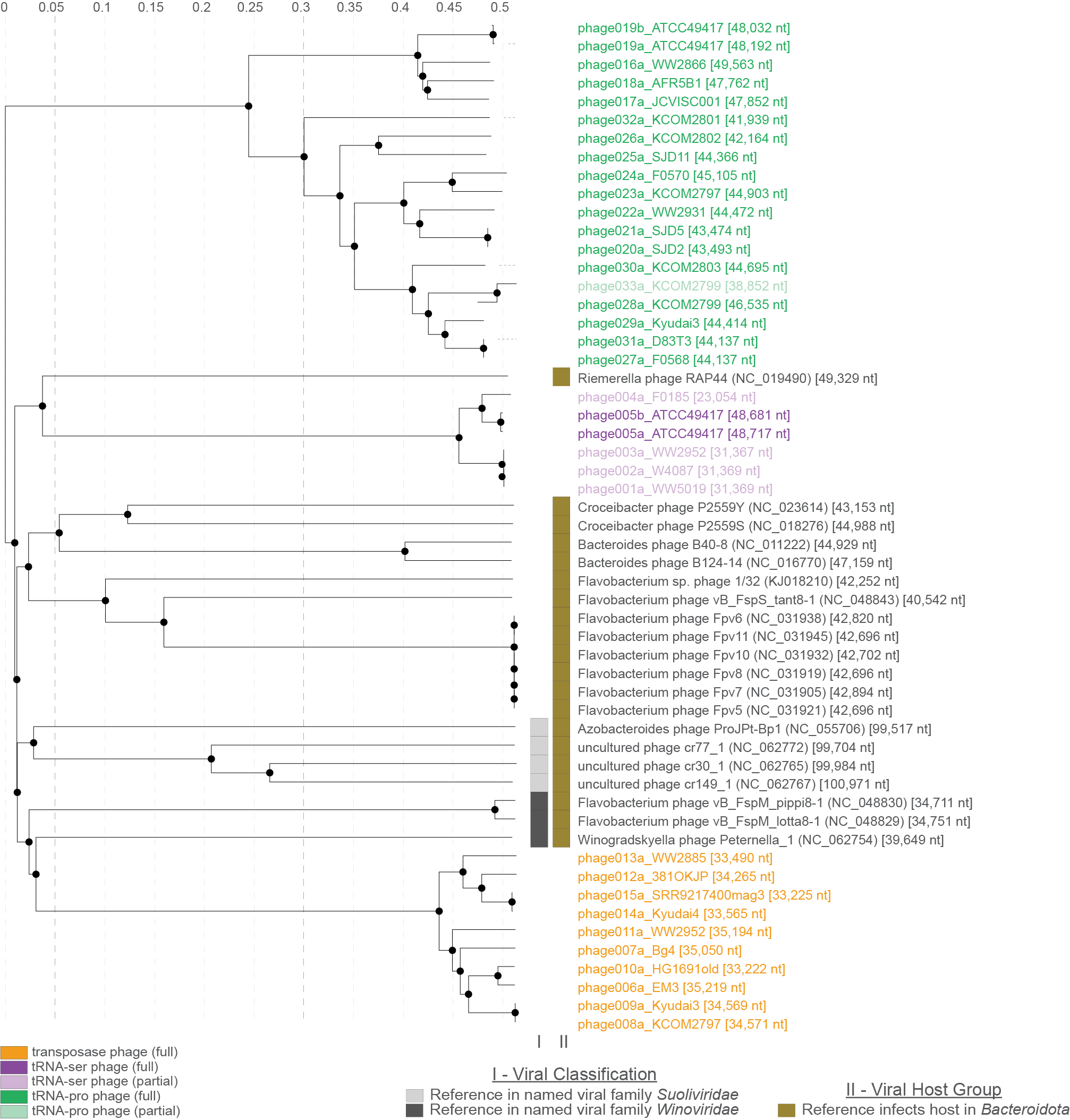

Supplement: Supplementary file 5 — Additional file 4: Supplementary Figure 4. Identification of nearest-neighbors of Porphyromonas gingivalis phages among reference phages, on the basis of ViPTree tBLASTx-based intergenomic distances. Placement of Pg phages among most sequence-similar reference phages in the 4,912 dsDNA Prokaryote-infecting viruses in the ViPTree [31] v3.5 Virus-Host DB [32] reference set, based on genome-wide tBLASTx-based sequence similarities. Pg phages are highlighted with labels colored corresponding to their insertion group type and completeness, reference phages in named families are indicated with boxes in shades of grey adjacent to their names (I), and reference phages infecting in the Bacteroidetes are indicated with a brown box adjacent to their names (II). All sequences included in this clade are reported in Supplementary Data 3. [file 40168_2023_1607_MOESM4_ESM.png]

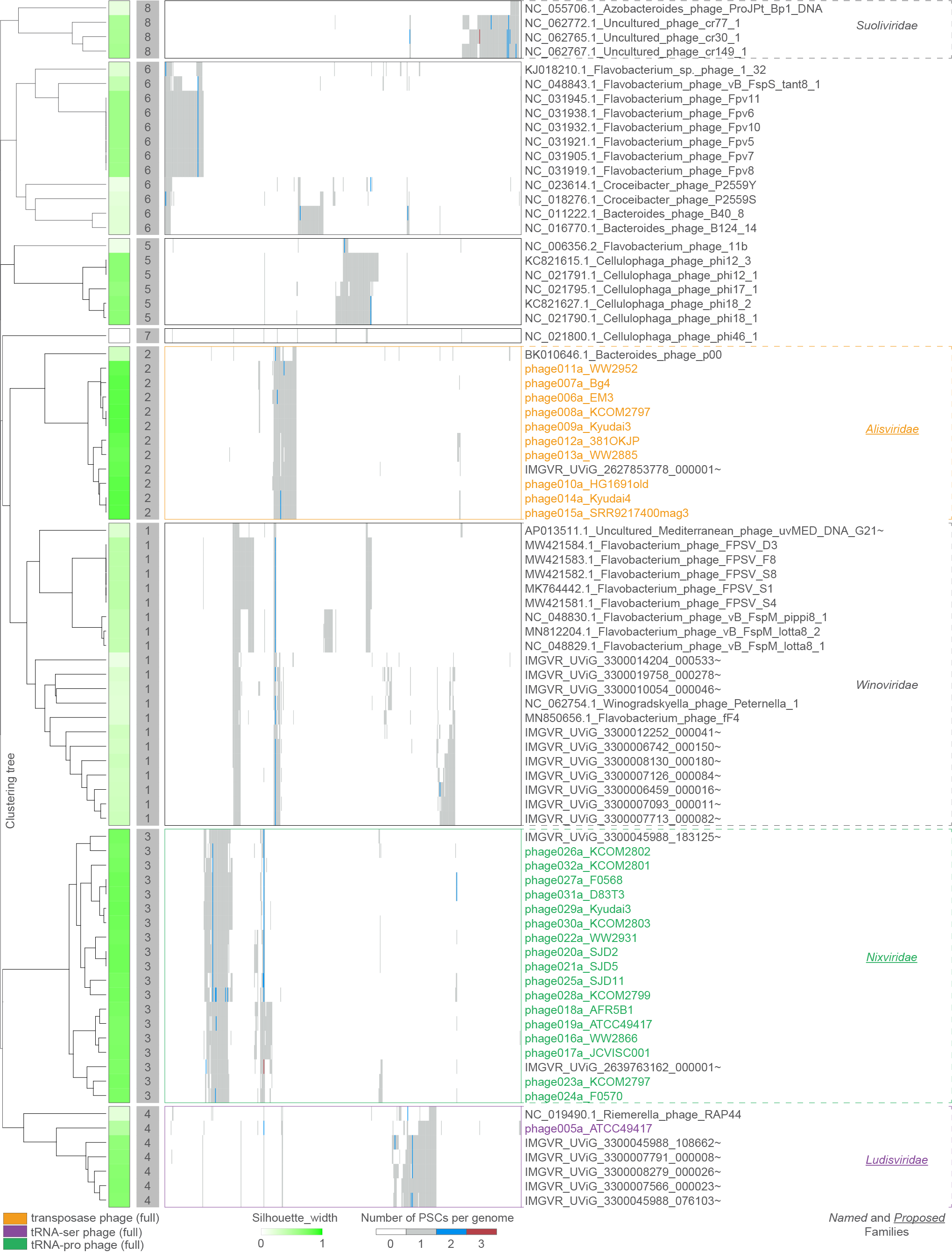

Supplement: Supplementary file 6 — Additional file 5: Supplementary Figure 5. Resolution of Porphyromonas gingivalis phages to three family level units, on the basis of VirClust Protein Super Cluster (PSC)-based intergenomic distances. The set of 82 phages included in this analysis was comprised of: all full-length Pg phages identified in this study (this excluded duplicates identified in alternate assemblies of the same Pg strain); all Porphyromonadaceae UViGs in IMG/VRv4 [39] assigned to the same vConTACT2 [30] Viral Cluster with the Pg phages, but not including those representing redundant geNomad [40] versions of the Pg phages; all reference phages identified in the ViPTree [31] placement tree as occurring within the same clade containing all Pg phages; all representatives of the closely-related viral family Winoviridae [36] identified in GenBank and the publication describing this group, as well as phages identified in the aforementioned publication as potentially related to the Winoviridae but lying outside the family (e.g. Bacteroides phage p00 and Cellulophaga phage phi46); all sequence accessions are reported in Supplementary Data 3. The VirClust [33] tree on the left reflects hierarchical clustering based on whole genome protein supercluster similarity; the silhouette width measures relatedness of a virus to other viruses within its own viral genome cluster (VGC) and to viruses outside of its VGC, with -1 indicating greatest similarity to viruses in other VGCs and 1 indicating greatest similarity to viruses within the same VGC (none <0, only range from 0 to 1 shown); the matrix represents all protein super clusters (PSCs) identified in the entire dataset (columns), with the number of PSCs per genome indicated by cell color. Pg phages are highlighted with leaf labels colored corresponding to insertion group type and completeness, clades identified as distinct family-level clusters by VirClust [33] are highlighted with dashed outlines, named and proposed families of phages are indicated in it [file 40168_2023_1607_MOESM5_ESM.png]

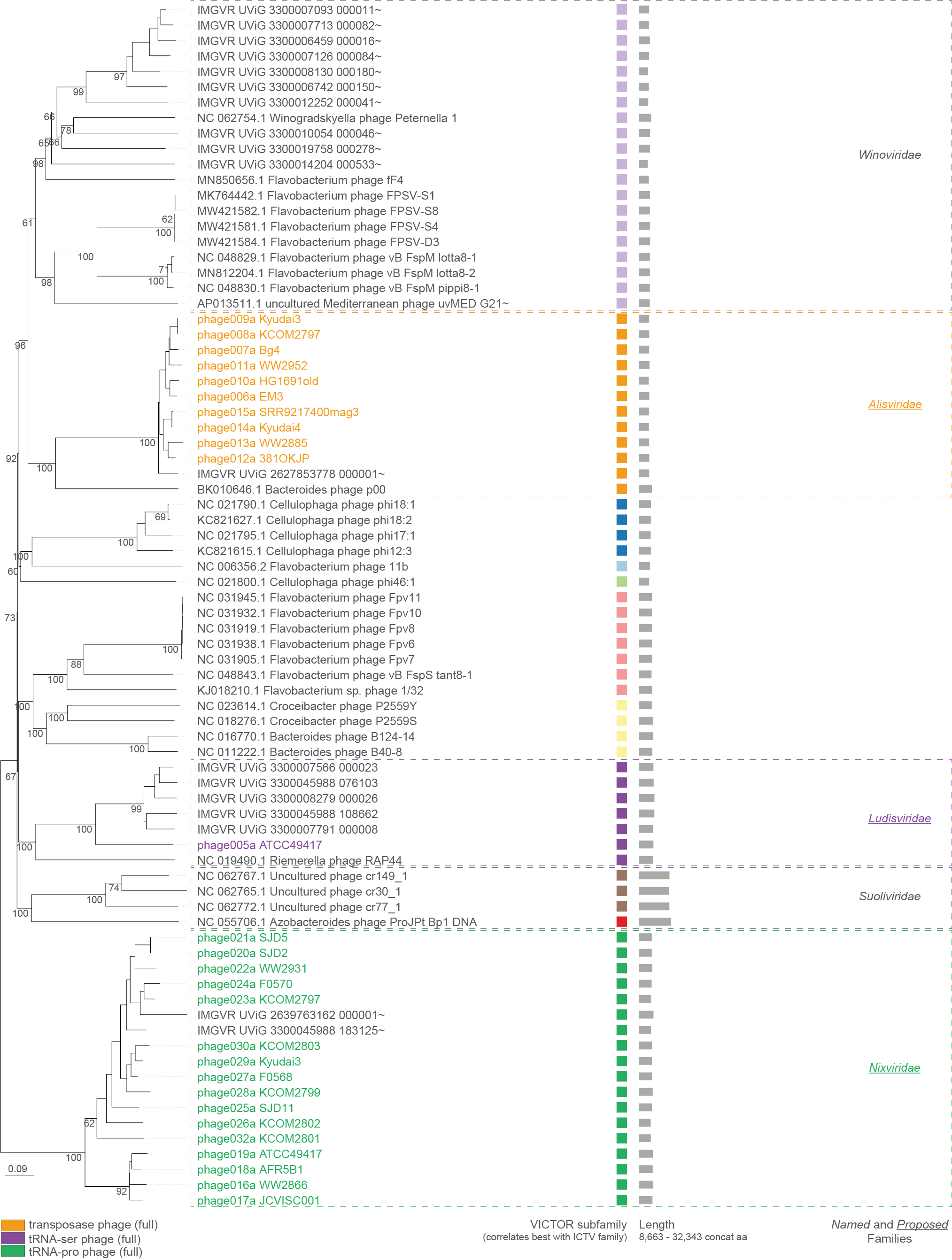

Supplement: Supplementary file 7 — Additional file 6: Supplementary Figure 6. Resolution of Porphyromonas gingivalis phages to three family level units, on the basis of VICTOR whole proteome intergenomic distances. The set of 82 phages included in this analysis was comprised of: all full-length Pg phages identified in this study (this excluded duplicates identified in alternate assemblies of the same Pg strain); all Porphyromonadaceae UViGs in IMG/VRv4 [39] assigned to the same vConTACT2 [30] Viral Cluster with the Pg phages, but not including those representing redundant geNomad [40] versions of the Pg phages; all reference phages identified in the ViPTree placement tree as occurring within the same clade containing all Pg phages; all representatives of the closely-related viral family Winoviridae [36] identified in GenBank and the publication describing this group, as well as phages identified in the aforementioned publication as potentially related to the Winoviridae but lying outside the family (e.g. Bacteroides phage p00 and Cellulophaga phage phi46); all sequence accessions are reported in Supplementary Data 3. The tree on the left reflects whole proteome similarity based on VICTOR [28] d6 (recommended for amino acid datasets) formula whole proteome distances, with branch supports based on 100 pseudo-bootstrap replicates; Pg phages are highlighted with leaf labels colored corresponding to insertion group type and completeness; clades identified as distinct subfamily-level clusters by VICTOR [28] (best corresponding to currently accepted thresholds for ICTV viral families) are indicated with colored boxes and highlighted with dashed outlines for named and proposed families of phages, indicated in italics and with underlines, respectively. [file 40168_2023_1607_MOESM6_ESM.png]

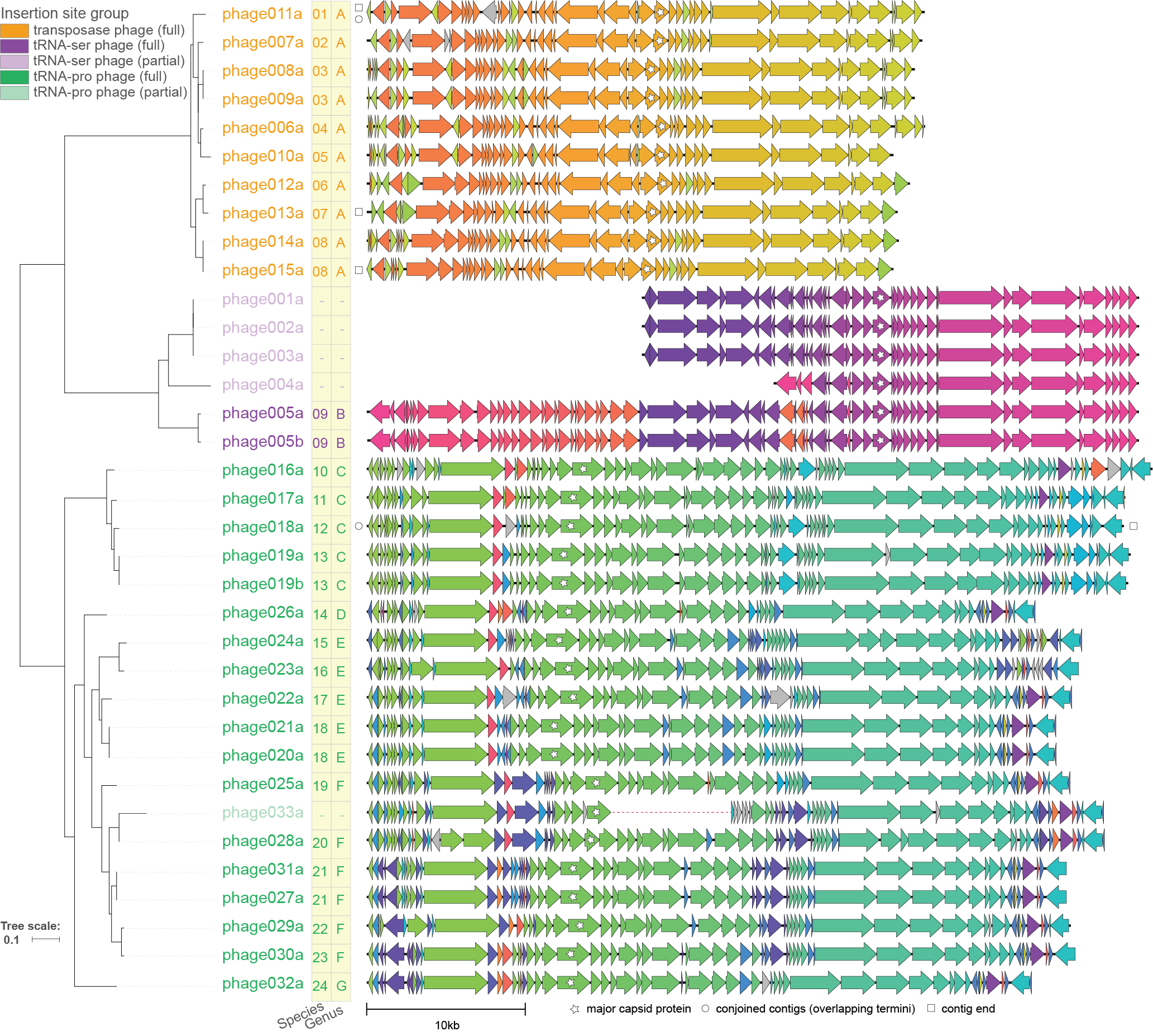

Supplement: Supplementary file 8 — Additional file 7: Supplementary Figure 7. Genome diagrams of Porphyromonas gingivalis phages show conservation of protein clusters. Pg phage phylogeny (30 full, 5 partial; names of full length phages are in saturated colors and partial phages are in lighter shades; “b” suffix indicates version of an “a” phage found in a different assembly of the Pg strain; midpoint-rooted tree based on whole genome nucleotide BLAST distance scaled by VICTOR [28] d0 formula, recommended for nucleic acid datasets) and genome diagram (generated using Clinker [42]) as shown in Fig. 2, with the exception that the predicted protein-coding genes (depicted as block arrows) are colored based on sequence similarity. Thus, highlighting the conservation of protein clusters and ordering among related Pg phages. Candidate genus- and species-level clusters are shown for full-length phages in the yellow bars. Three higher-order clades of phages defined by distinct insertion sites in host genomes (by full-length phages only) are highlighted by coloring of phage names (orange: transposition-based insertion; purple: tRNA-ser; green: tRNA-pro). White stars mark phage genome ends defined by contig ends, circles mark phage genomes identified in this work by joining contigs with overlapping termini, the dotted line in the middle of phage033a highlights that this phage was identified at the two termini of a bacterial contig assembly and is missing genes potentially due to an incomplete assembly. [file 40168_2023_1607_MOESM7_ESM.png]

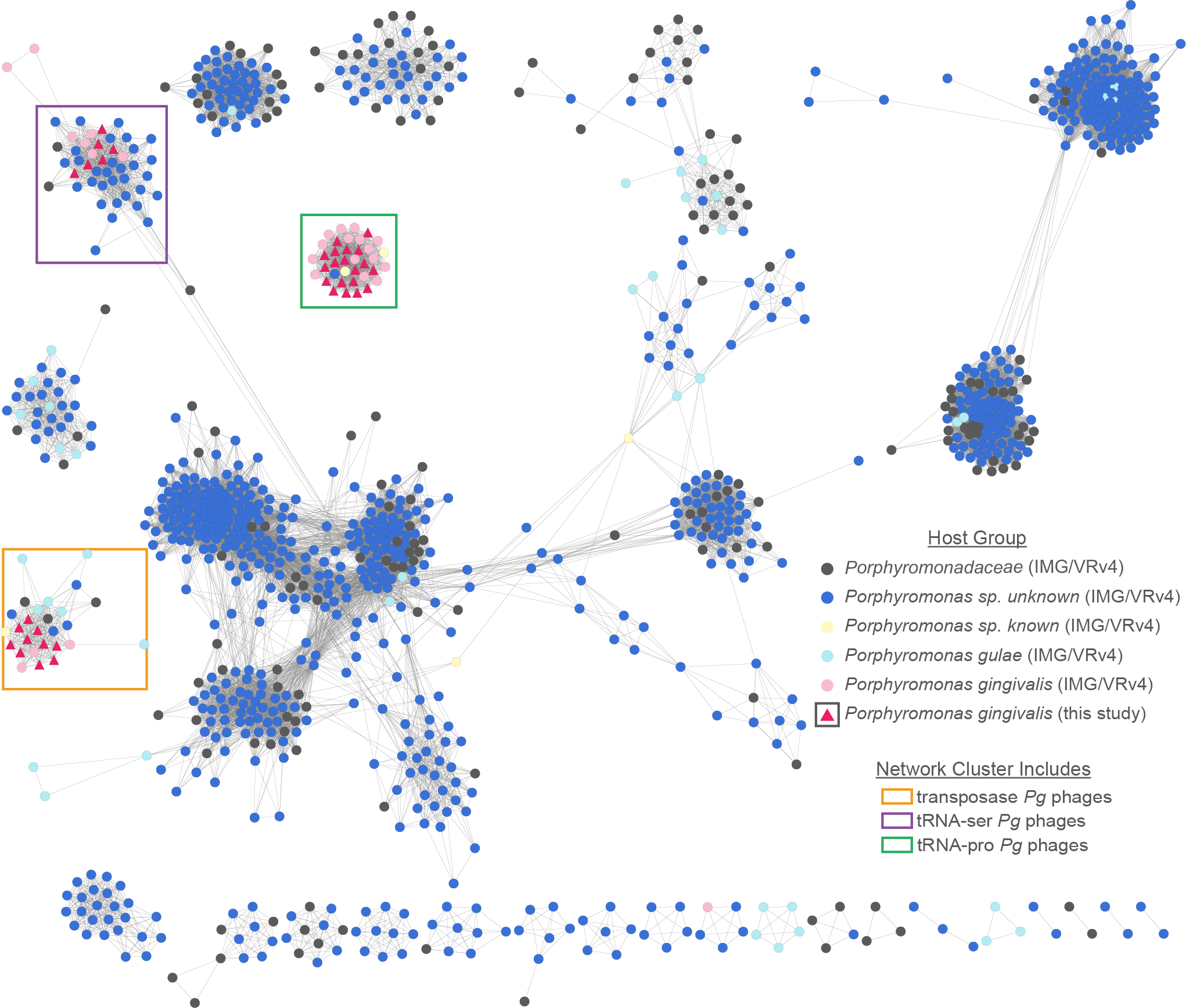

Supplement: Supplementary file 9 — Additional file 8: Supplementary Figure 8. Identification of relatives of Porphyromonas gingivalis phages among phages in genomic- and metagenomic-datasets in IMG/VRv4, and predicted to infect hosts in the Porphyromonadaceae. Network representation of vConTACT2 [30] whole proteome similarity among all Pg phages and 1,138 uncultivated viral genomes (UViGs) in IMG/VRv4 [39] known or predicted to infect hosts in the Porphyromonadaceae. Nodes represent viral genomes and are colored based on known or predicted host species, with triangles identifying Pg phages identified in this study. Network clusters containing Pg phages identified in this study are highlighted with colored boxes; note that vConTACT2 [30] defines cohesive Viral Clusters (VCs) that may contain only subsets of nodes appearing together in the same network cluster (see Supplementary Data 3). [file 40168_2023_1607_MOESM8_ESM.png]

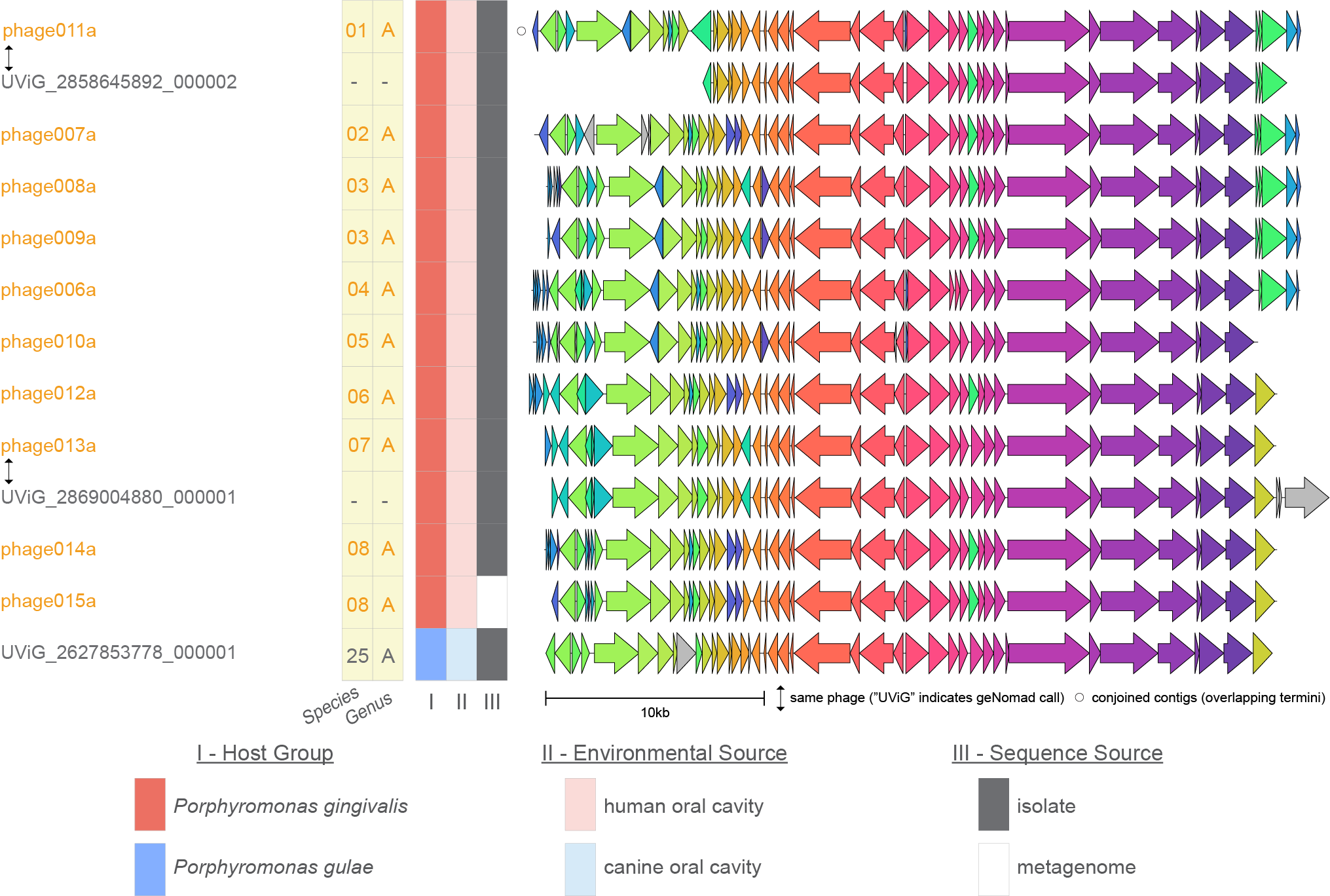

Supplement: Supplementary file 10 — Additional file 9: Supplementary Figure 9. Transposable Porphyromonas gingivalis phages identified in this study share synteny with UViGs. All uncultivated viral genomes (UViGs) reported in IMG/VRv4 [39] as predicted to infect Porphyromonadaceae were clustered with Pg phages on the basis of shared proteins using vConTACT2 [30]. All UViGs assigned to the same vConTACT2 [30] Viral Cluster as the transposable Pg phages are shown here and were aligned with the Pg phages using Clinker [42] v0.0.27, with ordering based on Pg phage only tree shown in Fig. 2 and placement of UViGs assisted by a whole genome distance tree generated using the VICTOR [28] d4 distance formula (recommended for datasets with numerous different length sequences) with nucleic acid input. In addition to the ten transposable Pg phages identified in this study, three UViGs are shown: two IMG/VRv4 [39] geNomad [40] pipeline versions of the same prophages (represented by double-headed arrows) and one prophage predicted in a Porphyromonas gulae genome. Also shown are the known or predicted phage host group (I), environmental source (II), and sequence source (III). The P. gulae UViG is predicted to represent a distinct species-level group within the same genus-level group as the Pg phages, as determined based on whole-genome nucleotide similarity with VIRIDIC [34]. [file 40168_2023_1607_MOESM9_ESM.png]

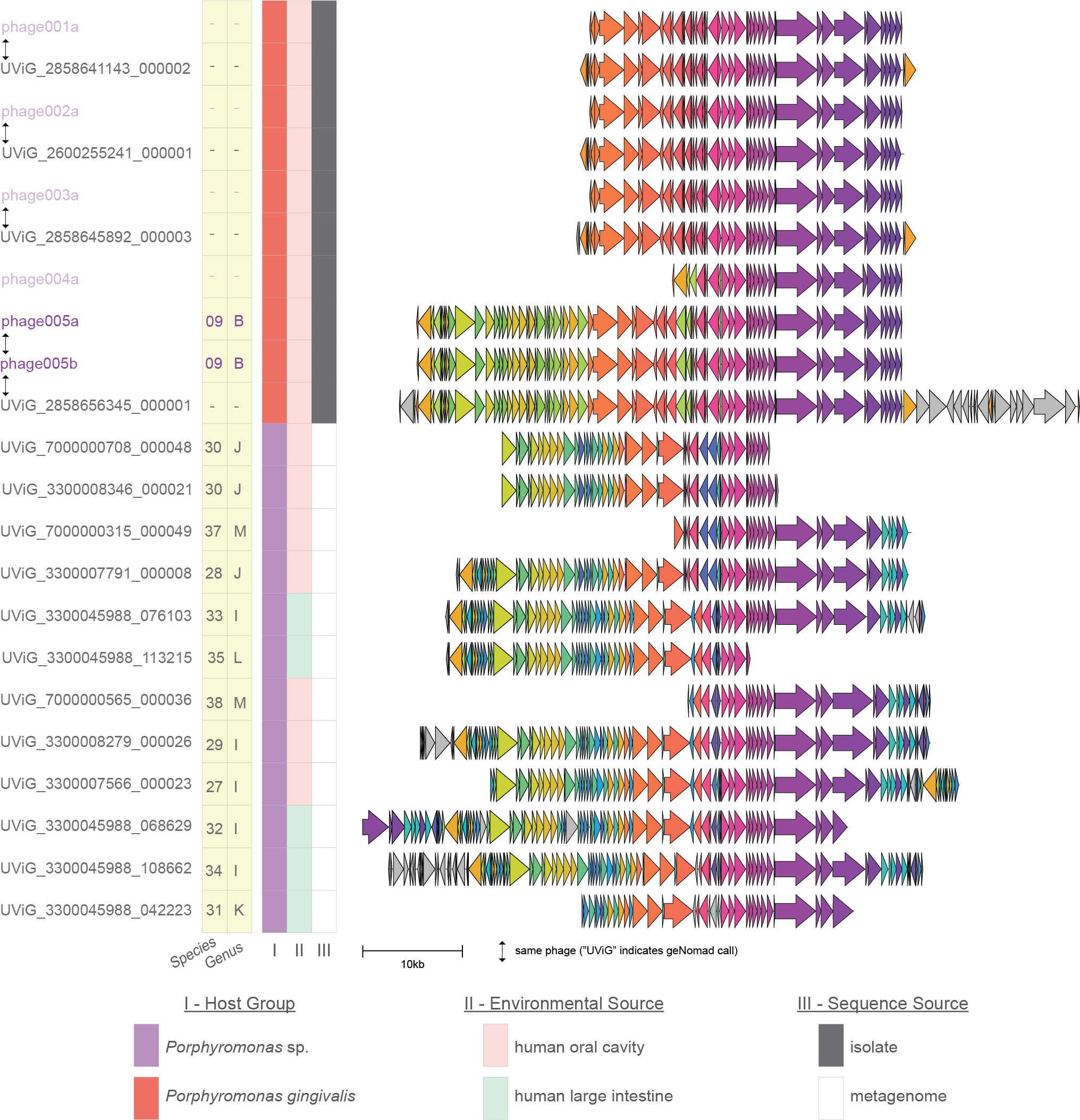

Supplement: Supplementary file 11 — Additional file 10: Supplementary Figure 10. Porphyromonas gingivalis phages with tRNA-serine gene insertion sites identified in this studying share synteny with UViGs. All uncultivated viral genomes (UViGs) reported in IMG/VRv4 [39] as predicted to infect Porphyromonadaceae were clustered with Pg phages on the basis of shared proteins using vConTACT2 [30]. All UViGs assigned to the same vConTACT2 [30] Viral Cluster as the tRNA-ser inserting Pg phages are shown here and were aligned with the Pg phages using Clinker [42] v0.0.27, with ordering based on Pg phage only tree shown in Fig. 2 and placement of UViGs assisted by a whole genome distance tree generated using the VICTOR [28] d4 distance formula (recommended for datasets with numerous different length sequences) with nucleic acid input. In addition to the five (partial- and full-length) tRNA-ser Pg phages identified in this study, 16 UViGs are shown: four IMG/VRv4 [39] geNomad [40] pipeline versions of the same prophages (represented by double-headed arrows) and 12 UViGs predicted from oral and intestinal metagenomes. Also shown are the known or predicted phage host group (I), environmental source (II), and sequence source (III). The Pg phages represent a distinct genus-level group from the UViGs identified in the metagenomic datasets, as determined based on whole-genome nucleotide similarity with VIRIDIC [34]. [file 40168_2023_1607_MOESM10_ESM.png]

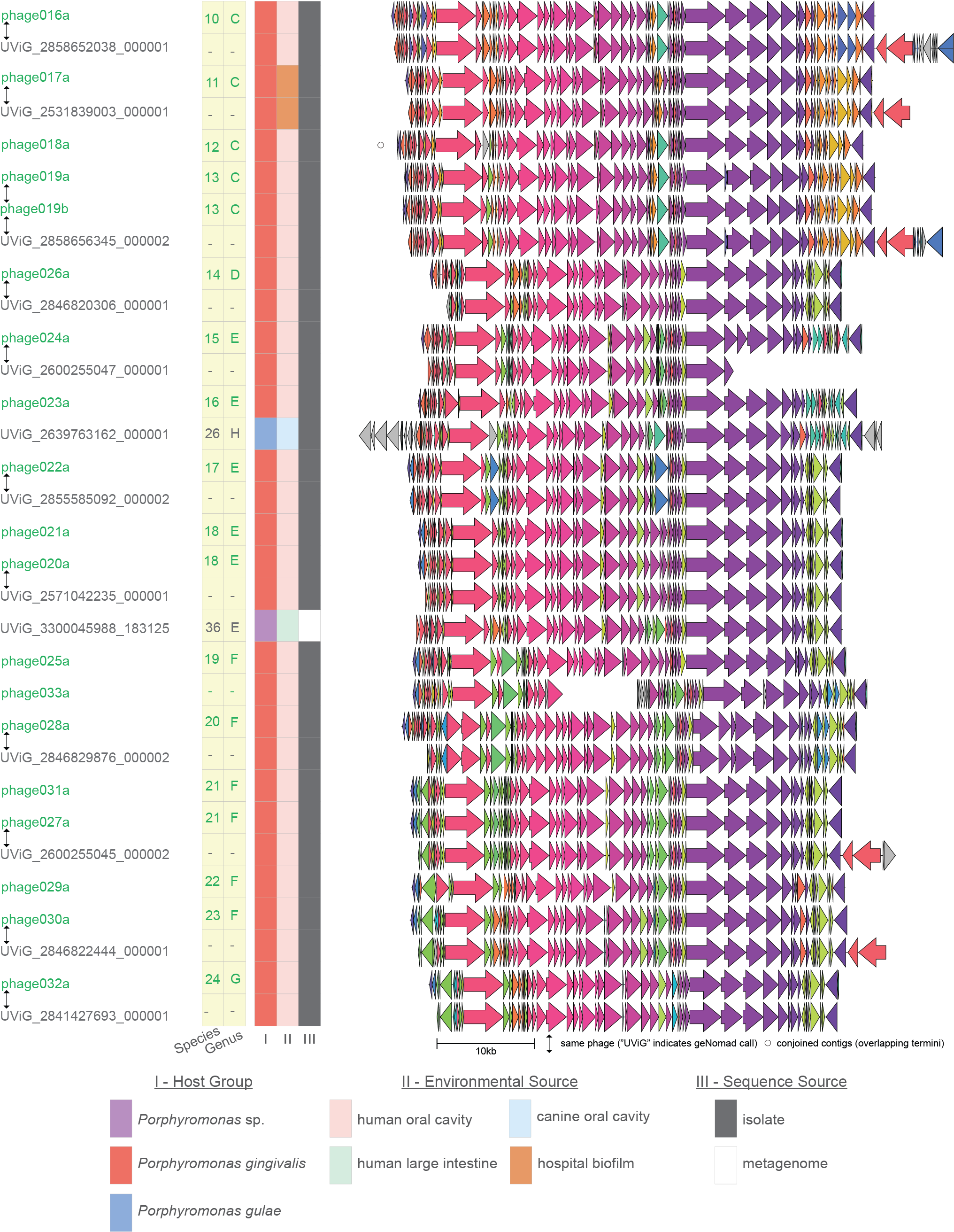

Supplement: Supplementary file 12 — Additional file 11: Supplementary Figure 11. Porphyromonas gingivalis phages with tRNA-proline gene insertion sites identified in this studying share synteny with UViGs. All uncultivated viral genomes (UViGs) reported in IMG/VRv4 [39] as predicted to infect Porphyromonadaceae were clustered with Pg phages on the basis of shared proteins using vConTACT2 [30]. All UViGs assigned to the same vConTACT2 [30] Viral Cluster as the tRNA-pro inserting Pg phages are shown here and were aligned with the Pg phages using Clinker [42] v0.0.27, with ordering based on Pg phage only tree shown in Fig. 2 and placement of UViGs assisted by a whole genome distance tree generated using the VICTOR [28] d4 distance formula (recommended for datasets with numerous different length sequences) with nucleic acid input. In addition to the 18 (partial- and full-length) tRNA-pro Pg phages identified in this study, 13 UViGs are shown: 11 IMG/VRv4 [39] geNomad [40] pipeline versions of the same prophages (represented by double-headed arrows), one predicted Porphyromonas gulae prophage, and one UViG from an intestinal metagenome. Also shown are the known or predicted phage host group (I), environmental source (II), and sequence source (III). Whereas the intestinal UViG is predicted to belong to the same genus as some of the Pg phages, the P. gulae prophage represents a distinct genus-level group, as determined based on whole-genome nucleotide similarity with VIRIDIC [34]. [file 40168_2023_1607_MOESM11_ESM.png]

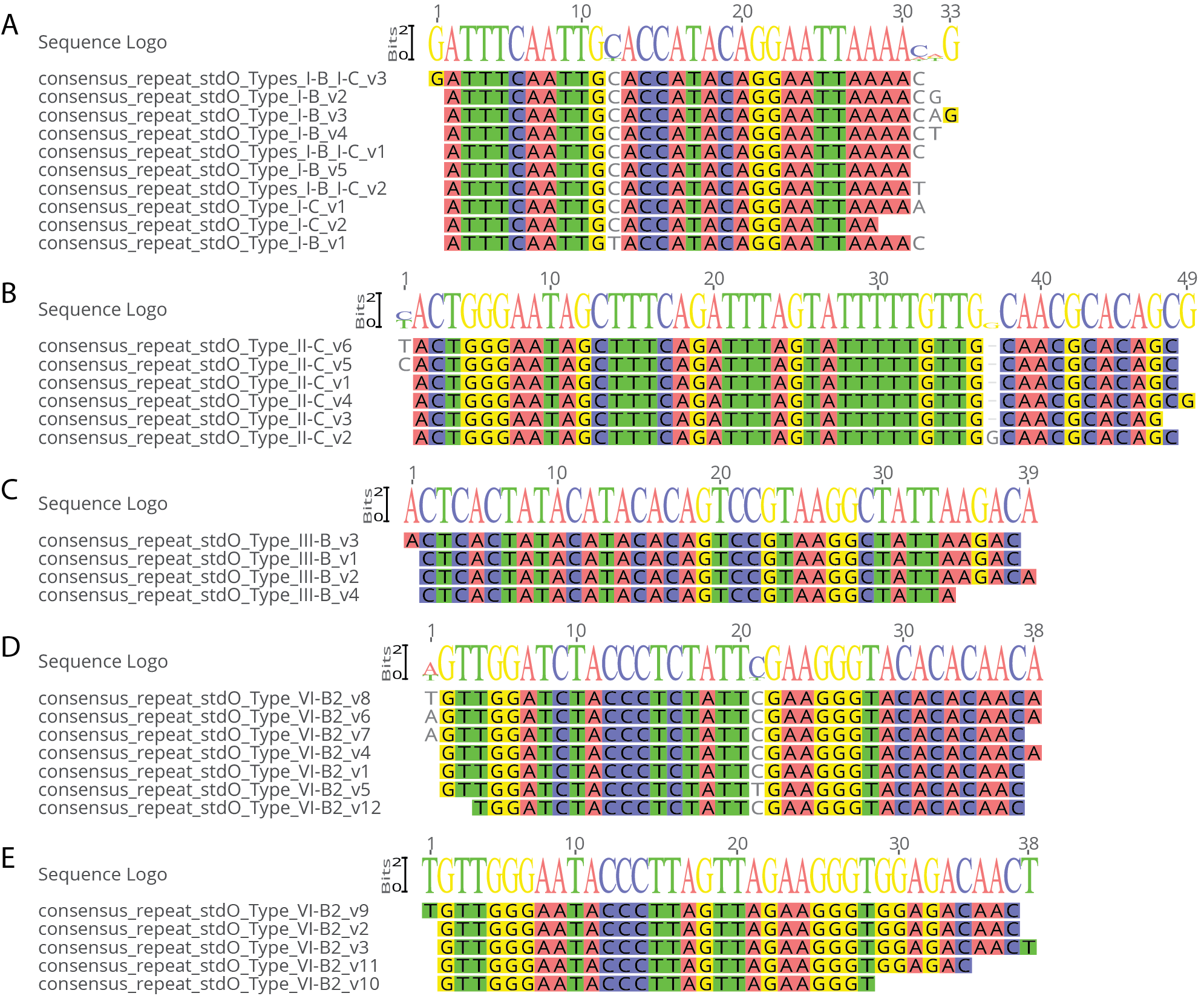

Supplement: Supplementary file 13 — Additional file 12: Supplementary Figure 12. Consensus sequences for repeats within individual Porphyromonas gingivalis CRISPR-Cas arrays show intermingling within some systems and sequence diversity within others. Type I-B and I-C arrays share common consensus repeats (A), whereas Type II-C (B) and Type III-B (C) arrays have distinct consensus repeats. Notably, for the Type VI-B arrays, there are two distinct groups of conserved repeats, one of these groups is associated with Type VI-B arrays that are part of the Pg core genome (D), whereas the other group (E) is associated with flexible Type VI-B systems. Consensus repeats shown are those from Pg_set79, in standardized orientation, and excluding repeats for which no CRISPR-Cas system type could be predicted (underlying data available in Supplementary Data 5). [file 40168_2023_1607_MOESM12_ESM.png]

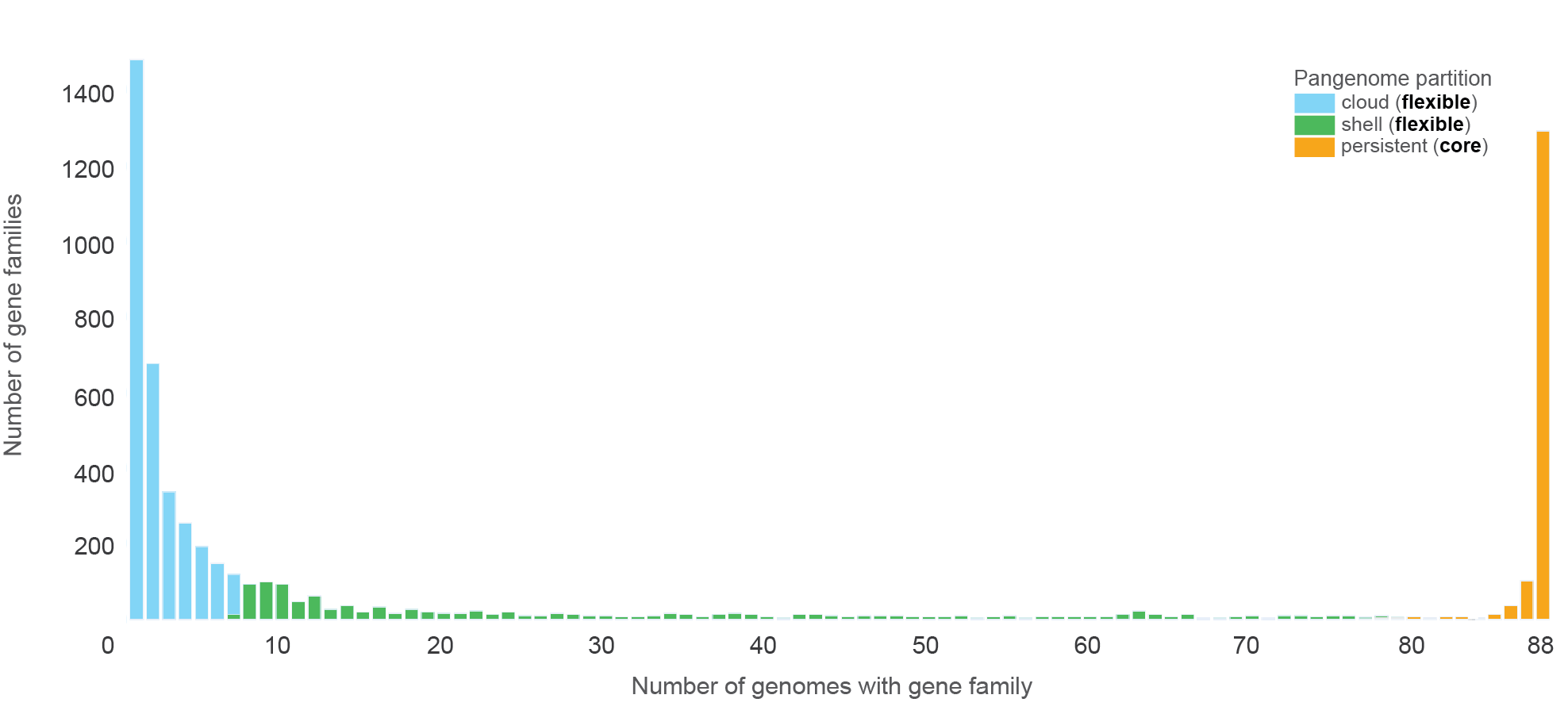

Supplement: Supplementary file 14 — Additional file 13: Supplementary Figure 13. Pangenomic partitioning of all 88 Porphyromonas gingivalis strains reveals an abundance of flexible genes. Plot indicates the number of gene families occurring in each of a given number of Pg genomes, from gene families that occur in only one genome to gene families that are found in all 88 (as predicted by PPanGGOLiN [77] using clustered proteins for Pg_set88). Light blue and green bars represent counts of gene families with “cloud” and “shell” designations by PPanGGOLiN [77] (combined and referred to in the text as making up the “flexible” pangenome), respectively, while orange bars represent “persistent” designations (referred to in the text as making up the “core” pangenome). [file 40168_2023_1607_MOESM13_ESM.png]

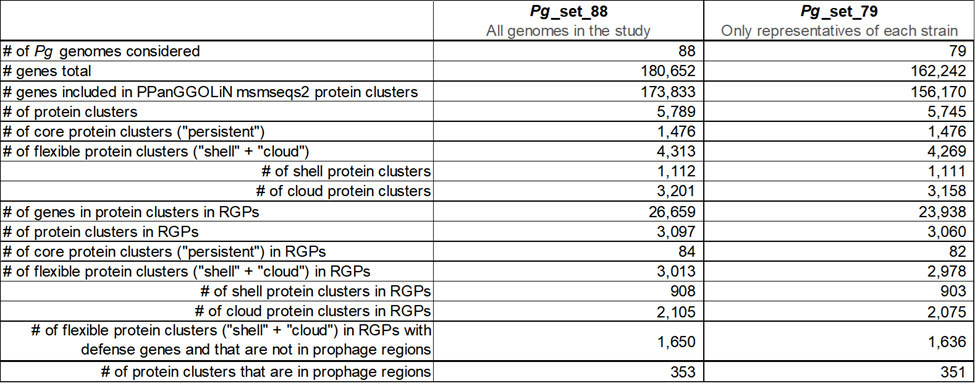

Supplement: Supplementary file 15 — Additional file 14: Supplementary Table 1. Summary information describing differences between sets of Pg genomes used in this study, with Pg_set_88 including all genomes and Pg_set_79 including only representatives of each strain to eliminate inflation of feature counts in various analyses resulting from inclusion of near-identical genomes. [file 40168_2023_1607_MOESM14_ESM.png]

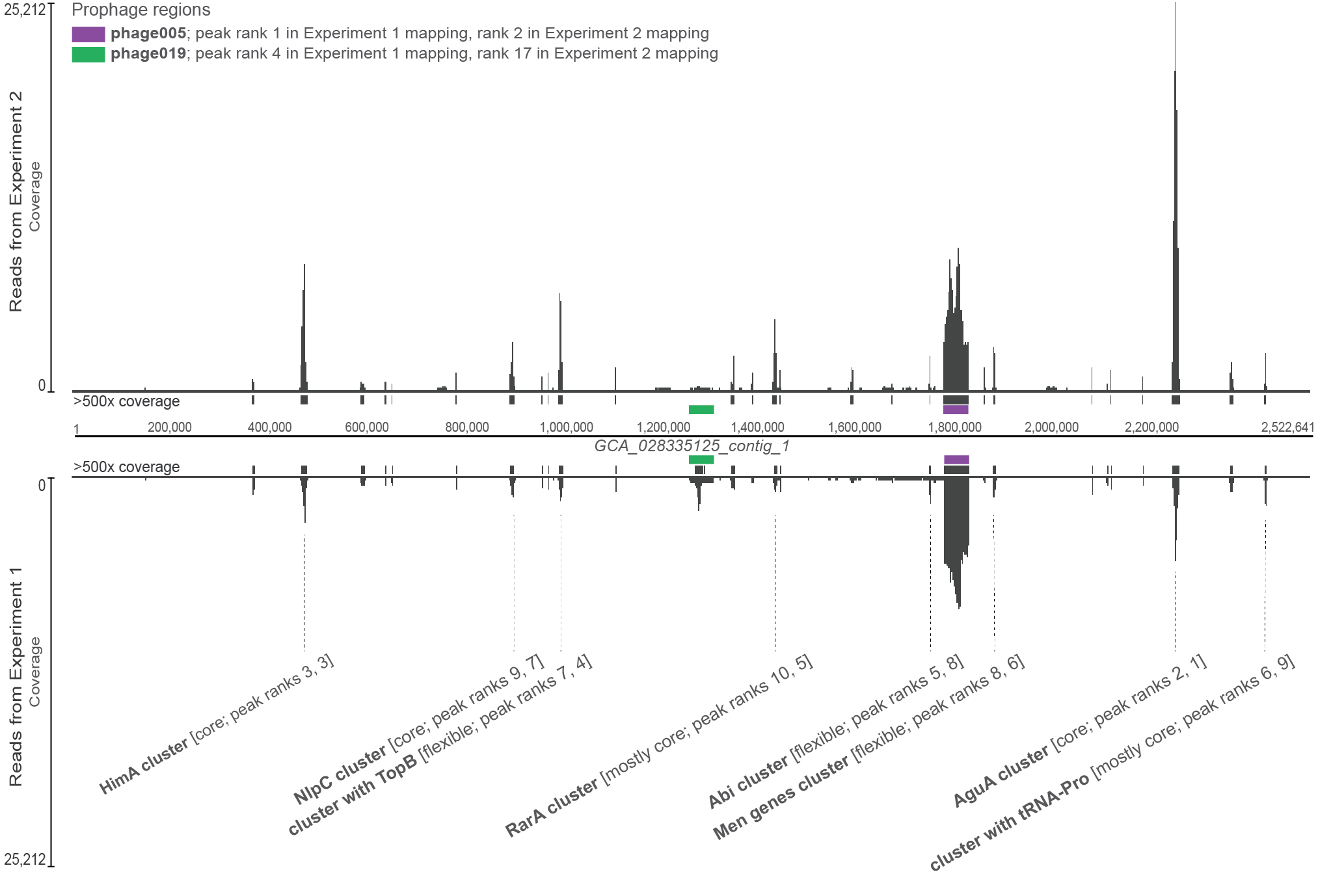

Supplement: Supplementary file 16 — Additional file 15: Supplementary Figure 14. Read mapping of filtered, nuclease-treated supernatants reveals the presence of protected, extracellular DNA in Porphyromonas gingivalis cultures. Mapping of Illumina sequencing reads from DNA extracted from cell-free, nuclease-treated, ultracentrifuged pellets of supernatants of ATCC 49417 Pg cultures. Bottom mapping represents reads from “Experiment 1”, which used supernatants from a 19-day old culture; an aliquot of the same culture at a younger age was used to obtain cell pellets from which assembly GCA_028993465 was produced. Top mapping represents reads from “Experiment 2”, which used supernatants from a 20-day old culture; an aliquot of the same culture at a younger age was used to obtain cell pellets from which assembly GCA_028335125 was produced. Both cultures were struck from glycerols originally derived from the same parent glycerol. Reads from both experiments were mapped onto the closed GCA_028335125 assembly and show coverage spikes along the genome (dark gray plots). Regions with greater than 500x coverage are marked by dark gray bars along the length of the reference, regions encoding phage005 and phage019 and marked by purple and green bars, respectively. Select additional peaks of high coverage are also shown, with clusters of elevated coverage named for the gene of highest coverage within the cluster or, where the peak gene is a hypothetical, with the name indicating another gene of known function nearby in the cluster. Mean coverage data for each protein-coding gene in the reference assembly is provided in Supplementary Data 11. [file 40168_2023_1607_MOESM15_ESM.png]
